# Heterologous prime-boost immunizations with chimpanzee adenoviral vectors elicit potent and protective immunity against SARS-CoV-2 infection

**DOI:** 10.1038/s41421-021-00360-4

**Published:** 2021-12-18

**Authors:** Jiaojiao Liu, Kun Xu, Man Xing, Yue Zhuo, Jingao Guo, Meng Du, Qi Wang, Yaling An, Jinhe Li, Ping Gao, Yihan Wang, Furong He, Yingying Guo, Mingxi Li, Yuchao Zhang, Linqi Zhang, George F. Gao, Lianpan Dai, Dongming Zhou

**Affiliations:** 1 Department of Pathogen Biology, School of Basic Medical Sciences, Tianjin Medical University, Tianjin, China; 2 Key Laboratory of Tropical Translational Medicine of Ministry of Education, School of Tropical Medicine and Laboratory Medicine, The First Affiliated Hospital, Hainan Medical University, Hainan, China; 3 Research Network of Immunity and Health (RNIH), Beijing Institutes of Life Science, Chinese Academy of Sciences, Beijing, China; 4 University of Chinese Academy of Sciences, Beijing, China; 5 Institut Pasteur of Shanghai, Chinese Academy of Sciences, Shanghai, China; 6 Savaid Medical School, University of Chinese Academy of Sciences, Beijing, China; 7 Comprehensive AIDS Research Center, Beijing Advanced Innovation Center for Structural Biology, School of Medicine and Vanke School of Public Health, Tsinghua University, Beijing, China; 8 CAS Key Laboratory of Tissue Microenvironment and Tumor, Shanghai Institute of Nutrition and Health, University of Chinese Academy of Sciences, Chinese Academy of Sciences, Shanghai, China; 9 School of Life Science, Fudan University, Shanghai, China; 10 CAS Key Laboratory of Pathogenic Microbiology and Immunology, Institute of Microbiology, Chinese Academy of Sciences, Beijing, China; 11 Chinese Center for Disease Control and Prevention (China CDC), Beijing, China; 12 Shanghai Public Health Clinical Center, Fudan University, Shanghai, China

**Keywords:** Immunology, Biological techniques

## Abstract

A safe and effective vaccine for severe acute respiratory syndrome coronavirus 2 (SARS-CoV-2) is urgently needed to tackle the COVID-19 global pandemic. Here, we describe the development of chimpanzee adenovirus serotypes 6 and 68 (AdC6 and AdC68) vector-based vaccine candidates expressing the full-length transmembrane spike glycoprotein. We assessed the vaccine immunogenicity, protective efficacy, and immune cell profiles using single-cell RNA sequencing in mice. Mice were vaccinated via the intramuscular route with the two vaccine candidates using prime-only regimens or heterologous prime-boost regimens. Both chimpanzee adenovirus-based vaccines elicited strong and long-term antibody and T cell responses, balanced Th1/Th2 cell responses, robust germinal center responses, and provided effective protection against SARS-CoV-2 infection in mouse lungs. Strikingly, we found that heterologous prime-boost immunization induced higher titers of protective antibodies, and more spike-specific memory CD8^+^ T cells in mice. Potent neutralizing antibodies produced against the highly transmissible SARS-CoV-2 variants B.1.1.7 lineage (also known as N501Y.V1) and B.1.351 lineage (also known as N501Y.V2) were detectable in mouse sera over 6 months after prime immunization. Our results demonstrate that the heterologous prime-boost strategy with chimpanzee adenovirus-based vaccines is promising for further development to prevent SARS-CoV-2 infection.

## Introduction

As witnessed over the last 2 years, the rapid and global expansion of the coronavirus disease 2019 (COVID-19) pandemic, caused by severe acute respiratory syndrome coronavirus 2 (SARS-CoV-2)^1^, is a continued crisis affecting public health and the global economy. Chinese scientists first published the whole-genome sequence of SARS-CoV-2^2^, and ever since, multiple research programs and vaccine industries have participated in vaccine development. To date, several manufacturers have successfully developed COVID-19 vaccines based on various platforms and strategies, and some of these vaccines have been authorized or approved for emergency use in some countries, with several more in different stages of clinical trials^3^. Additionally, replication-deficient adenoviral vector technology has been employed to generate candidate vaccines, such as the Ad5-vectored COVID-19 vaccine developed by CanSino Biologics’ (Tianjin, China)^4^, vaccines based on a rare human adenovirus serotype Ad26 (Johnson & Johnson)^5^, the chimpanzee adenovirus ChAdOX1 (AstraZeneca/Oxford University)^6^, and a heterologous prime-boost strategy using human adenoviral vectors Ad5 and Ad26 in Russia^7^. All adenoviral-vectored COVID-19 vaccines mentioned above showed acceptable safety, tolerability, and immunogenicity.

Spike structural protein, which embeds in the viral surface envelope, is the main protein used as a target in COVID-19 vaccines, and the receptor-binding domain (RBD) of spike is the major target for neutralizing antibodies that interfere with viral receptor binding^8^. However, the highly transmissible SARS-CoV-2 variants B.1.1.7 lineage (also known as N501Y.V1 or VOC-202012/01) and B.1.351 lineage (also known as N501Y.V2), discovered and reported in the United Kingdom and South Africa, respectively, carrying mutations in the RBD of spike have led to wide concerns. Poor neutralizing potency against B.1.351 variant was observed in specimens obtained from participants in clinical trials of several COVID-19 vaccines^9,10^. Thus, as new mutations are constantly being reported, eliciting higher and broader protective immune responses is urgent for the development of COVID-19 vaccines, and a heterologous prime-boost strategy based on existing vaccine candidates may be a fast, safe, and economical way to achieve this goal.

In this study, we generated novel SARS-CoV-2 vaccines based on chimpanzee adenovirus serotypes 6 (also called simian adenovirus serotype 23, SAd-V23) and 68 (also called SAd-V25) vectors, both from family E, but two different serotypes, by incorporating the full-length spike protein. These two vectors have already been investigated in preclinical or clinical research and proved to be safe and efficient^11–13^. The AdC68 vector expressing the full-length spike protein of MERS-CoV, which is closely related to SARS-CoV-2, was demonstrated to induce strong and long-lasting immune responds^14^. An Ebola vaccine based on this vector also proved its effectiveness in a nonhuman primate model^12^. To elicit long-term and strong immune responses, we compared the different vaccination regimens, including prime-only regimens and heterologous prime-boost regimens, in two mouse strains. We then detected the neutralizing ability against the original Wuhan-Hu-1 strain of pseudo- or live virus and two predominant variants of the pseudovirus. Subsequently, protection efficacy against live virus was tested using a mouse model expressing human angiotensin-converting enzyme 2 (hACE2) in the lung, which has been reported as an effective model^15^. Combined with T/B cell immune responses and single-cell RNA sequencing (scRNA-seq), we investigated the best vaccine immunization strategy and provided novel insights into SARS-CoV-2 vaccine development.

## Results

### Vaccine characterization and long-term immune responses in mice

AdC6 and AdC68 vector-based vaccines expressed the full-length SARS-CoV-2 spike protein. The codon-optimized sequence of the spike gene was cloned into the E1 deletion region of the AdC68 and AdC6 genomes, respectively (Supplementary Fig. S1a). Western blot analyses confirmed the dose-dependent expression of spike proteins in HEK293 cell lysates (Supplementary Fig. S1b). Five 6–8-week-old female C57BL/6 mice per group were vaccinated using a prime-only regimen (AdC6-S and AdC68-S, respectively, day 0), or a heterologous prime-boost regimen (AdC6-S + AdC68-S and AdC68-S + AdC6-S, respectively, day 0 and day 28) intramuscularly (i.m.) with 2 × 10^10^ viral particles (vp). As controls, five animals per group were vaccinated using the same regimens and routes, with the same dose of AdC6-empty and AdC68-empty.

**Fig. 1:**
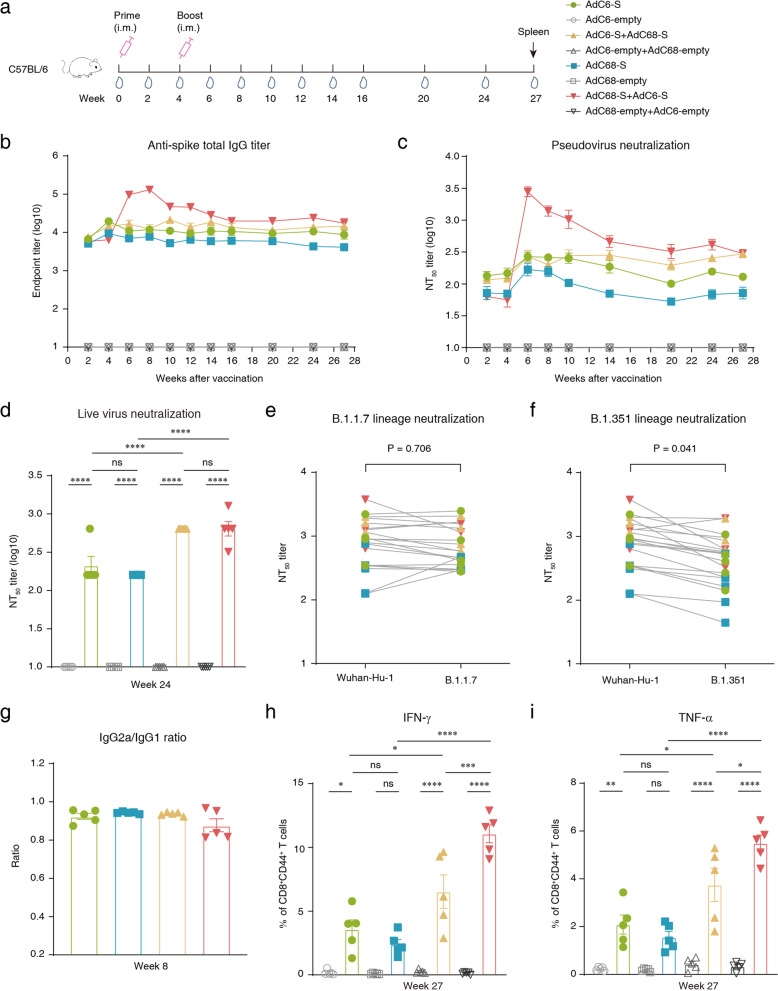
Long-term immune responses in immunized C57BL/6 mice. **a** Schedule of chimpanzee adenoviral vaccine immunization and bleeding strategies in female C57BL/6 mice (6–8-week-old). Eight groups (*n* = 5) of C57BL/6 mice were i.m. injected with 2 × 10^10^ vp viruses. Serum samples were then collected at different time points. **b** Kinetics of spike-specific total IgG reciprocal endpoint titers (log10) were measured within 27 weeks of initial vaccination. Total IgG immune responses were measured using ELISA. **c** Kinetics of SARS-CoV-2 pseudovirus neutralizing antibody NT_50_ titers (log10) were measured within 27 weeks after the first vaccination. **d** Measurement of live SARS-CoV-2 virus neutralizing antibody NT_50_ titers of sera collected at week 24. **e** Neutralization of variant B.1.1.7 SARS-CoV-2 pseudovirus in serum samples obtained at week 27 post initial immunization. Unpaired *t*-test was used for analysis. **f** Neutralization of variant B.1.351 SARS-CoV-2 pseudovirus in serum samples collected at week 27 after the first vaccination (unpaired *t*-test, *P* = 0.041). **g** Endpoint titer ratios of IgG2a to IgG1 were calculated. Mouse sera were collected at week 8, and subtypes IgGs were assessed by ELISA. Sera of mice immunized with AdC6-empty and AdC68-empty were not calculated, as the endpoint titer did not reach the lower limit of detection (LLOD). **h**, **i** Intracellular cytokine staining was performed in mouse spleen to assess memory T cells at week 27, and cytokines IFN-γ and TNF-α were detected. All data were shown as means ± SEM. *P*-values were analyzed with one-way ANOVA (^ns^*P* ≥ 0.05; *P < 0.05; **P < 0.01; ***P < 0.001; ****P < 0.0001).

To assess the long-term humoral immune response in mice, sera were collected every two weeks after initial immunization (Fig. 1a). Enzyme-linked immunosorbent assay (ELISA) was used for testing spike-specific binding antibodies, and a pseudo- and live virus neutralization assay was used to measure the neutralizing antibody responses. The binding and neutralizing antibody responses were both developed as early as 14 days after the first administration of the two vaccine candidates, with endpoint IgG titers of 7108 (geometric mean titer, GMT; AdC6-S) and 5489 (GMT, AdC68-S), and pseudovirus-based neutralizing titer 50 (NT_50_) of 125 (GMT, AdC6-S) and 67(GMT, AdC68-S). The antibody titers were slightly increased by week 4 (Fig. 1b, c). After the heterologous boost, the antibody titers of prime-boost groups showed an increase, and AdC68-S + AdC6-S group raised dramatically (Fig. 1b, c). All groups maintained high antibody titers within 27 weeks after priming immunization, and heterologous prime-boost strategies induced a stronger humoral immune response than others (Fig. 1b, c).

Live virus-neutralizing antibody titers in mouse serum samples collected at week 24 were then tested, and we found that the prime-boost regimen induced a stronger humoral immune response compared to that induced by the prime-only regimen. The NT_50_ titers of live virus-neutralizing antibodies in the AdC68-S + AdC6-S (GMT = 640) and AdC6-S + AdC68-S (GMT = 640) groups were 4- and 3-fold higher than those in the AdC68-S (GMT = 160) and AdC6-S groups (GMT = 211), respectively (Fig. 1d). Titers of pseudovirus neutralizing antibodies correlated with both anti-spike total IgG titers and live virus-neutralizing antibody titers (*R* = 0.9568, *P* < 0.0001 and *R* = 0.9760, *P* < 0.0001, respectively) (Supplementary Fig. S1c, d). Moreover, we used pseudoviruses bearing the full-length spike protein from the original Wuhan-Hu-1 SARS-CoV-2 strain, as well as variants of the B.1.1.7 and B.1.351 lineages to assess the neutralizing antibody titers in mouse serum samples, which were collected at week 27. Consistent with a previous report^9,10^, we found that the B.1.1.7 variant had no significant effect on neutralization (Fig. 1e). In contrast, we observed that neutralization of the B.1.351 variant was approximately two times weaker than that of the original SARS-CoV-2 Wuhan-Hu-1 strain (unpaired *t*-test, *P* = 0.041) (Fig. 1f). The GMT of the AdC6-S, AdC68-S, AdC6-S + AdC68-S, and AdC68-S + AdC6-S groups were 740, 287, 1147, and 1501 for the original Wuhan-Hu-1 strain; 617, 310, 1003, and 1181 for B.1.1.7 variant; and 387, 173, 938, and 615 for B.1.351 variant; respectively. In general, neutralizing antibody titers of sera against two variant pseudoviruses were largely preserved, and mice in the prime-boost groups developed a more robust antibody response over half a year after the prime.

Spike protein-specific IgG1, IgG2a, and IgG2b antibody titers were assessed using ELISA. Among these antibody isotypes, IgG1 and IgG2a have been associated with T cell response, but IgG2b is usually considered to be part of the T cell-independent response^16^. Both AdC6-S and AdC68-S vaccines elicited robust IgG1, IgG2a, and IgG2b antibody isotype responses within two weeks of priming immunization (Supplementary Fig. S2a–c). After boosting vaccination, higher titers of these three antibody isotypes were measured in the AdC68-S + AdC6-S group (Supplementary Fig. S2d–f). In addition, all three IgG subtypes maintained high titers in vaccinated mice at week 24 (Supplementary Fig. S2g–i). To evaluate the balance of Th1 and Th2 responses in vaccinated mice, the IgG2a/IgG1 ratio was calculated. The ratios of all vaccinated groups were 0.8–1.0, indicating a balanced Th1/Th2 response (Fig. 1g and Supplementary Fig. S1e, f). We also directly detected cytokine patterns in vaccine-induced memory T cells in mouse spleens by intracellular cytokine staining and ex vivo IFN-γ enzyme-linked immunosorbent spot assay (ELISpot), up to ~7 months after the initial vaccination. More IFN-γ- and TNF-α-secreted CD8^+^ memory T cells (as measured by CD44 expression on CD8^+^ T cells) were detected in two prime-boost groups after stimulation with the S1 peptide pool (Fig. 1h, i), with little IL-2 secretion (Supplementary Fig. S1g). Besides, more spots were observed in prime-boost groups by IFN-γ ELISpot assay (Supplementary Fig. S1h), indicating that boosting immunization is necessary for enhancing T cell responses.

### Vaccine-induced T cell responses in mice

**Fig. 2:**
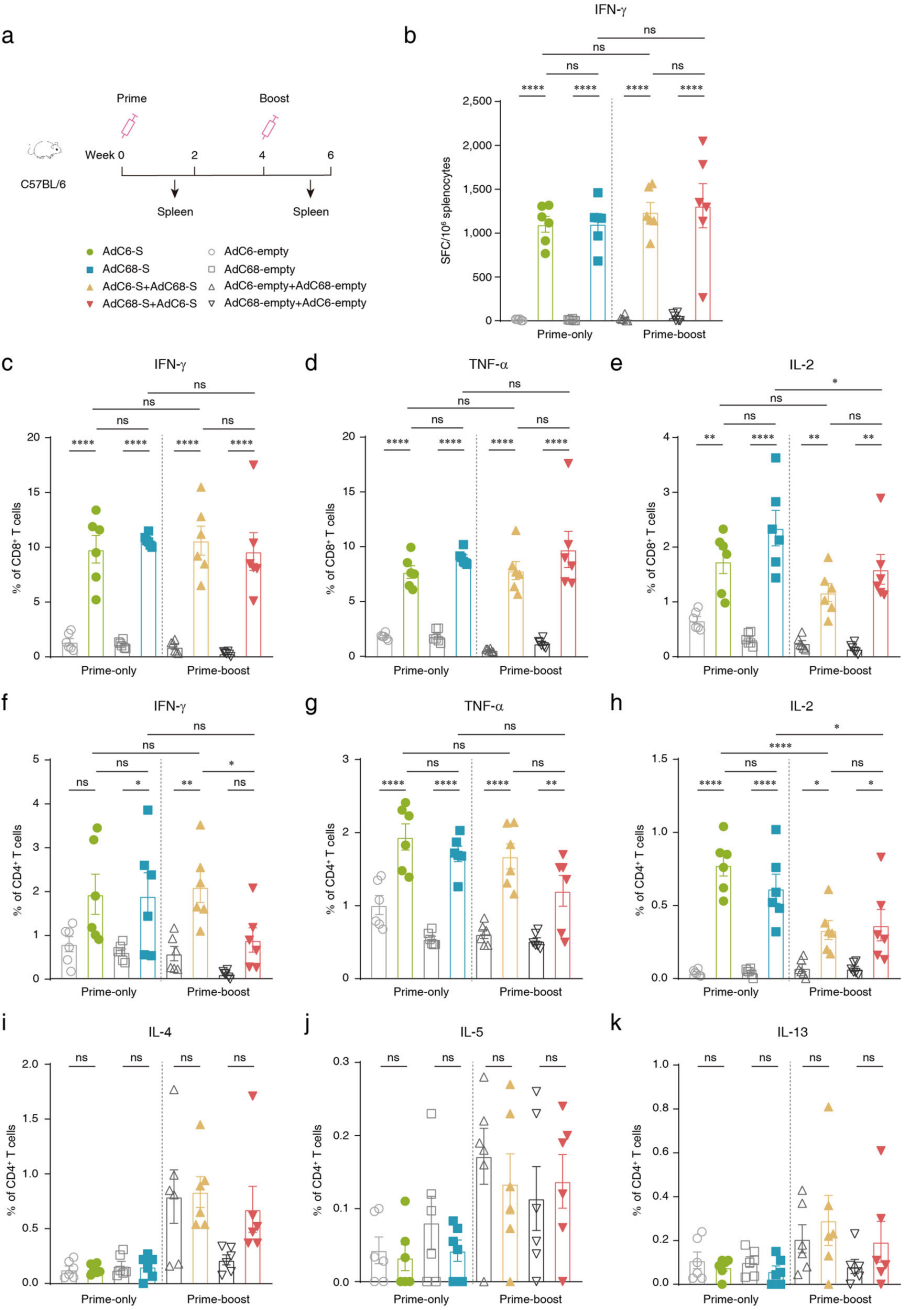
Cellular immune responses in vaccinated C57BL/6 mice. **a** Schedule of chimpanzee adenoviral vaccine immunization and cell sorting strategies in female C57BL/6 mice (6–8-week-old). Eight groups (*n* = 6) of C57BL/6 mice were i.m. injected with 2 × 10^10^ vp viruses. Splenocytes were collected on day 10 post priming and boosting immunization, respectively. **b** ELISpot assay was performed to measure the IFN-γ secretion of splenocytes after S1 peptide pool stimulation in vaccine-immunized mice. Cells were harvested on day 10 post vaccination. **c**–**e** Percentage of CD8^+^ cytotoxic T lymphocytes expressing IFN-γ, TNF-α, and IL-2 in response to the S1 peptide pool on day 10 post immunization. **f**–**h** Percentage of CD4^+^ helper T cells expressing IFN-γ, TNF-α, and IL-2 in response to the S1 peptide pool on day 10 post immunization. **i**–**k** Percentage of CD4^+^ helper T cells expressing IL-4, IL-5, and IL-13 in response to the S1 peptide pool on day 10 post immunization. All data were shown as means ± SEM. *P-*values were analyzed with one-way ANOVA (^ns^*P* ≥ 0.05; *P < 0.05; **P < 0.01; ***P < 0.001; ****P < 0.0001).

Six C57BL/6 mice per group were immunized with vaccines using a prime-only or prime-boost regimen, and T cell responses were characterized on day 10 after priming or booster vaccination, respectively. Splenocytes were harvested and stimulated with two overlapping 15-mer peptide pools representing the S1 and S2 subunits of the spike glycoprotein of SARS-CoV-2, respectively, and detected by using ELISpot assay and intracellular cytokine staining (Fig. 2a). A great deal of IFN-γ-secreted T cells were detected after stimulation with the S1 peptide pool, and the magnitude of the response had no variation between the prime-only regimen and prime-boost regimen (Fig. 2b), but not in IFN-γ ELISpot assay stimulated with S2 peptide pool (Supplementary Fig. S3a).

CD4^+^ and CD8^+^ T cell responses in the animals were assessed using flow cytometric analyses. All mice administered with two vaccine candidates via single-dose injection or heterologous prime-boost vaccination strategies induced robust cytotoxic T lymphocytes (CTL) and Th1 cell responses, detected as IFN-γ, TNF-α, and IL-2 production in splenic lymphocytes and stimulated with the S1 peptide pool on day 10 after the first and second injections, respectively (Fig. 2c–h). In addition, no substantial cytokine production was detected in cells stimulated with the S2 peptide pool (Supplementary Fig. S3b–g). Furthermore, Th2-based responses were also assessed using intracellular cytokine staining, detected as IL-4, IL-5, and IL-13 expression, while no Th2 cytokine-secreted cells were detected (Fig. 2i–k).

### Protection efficacy in mice

**Fig. 3:**
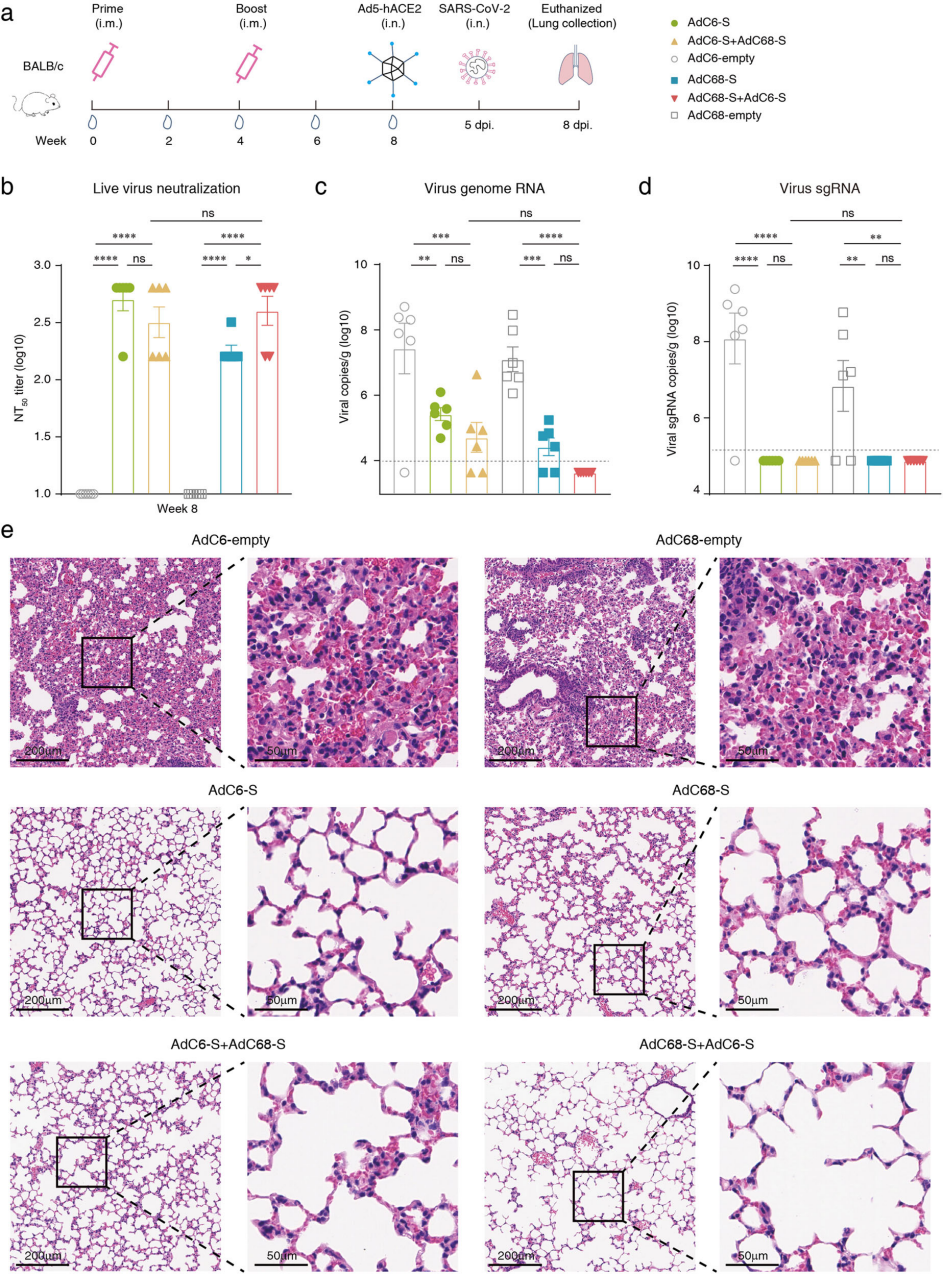
Immunogenicity and protective efficacy in BALB/c mice. **a** Schematics of vaccine immunization and challenge in female BALB/c mice (6–8-week-old). Mice (*n* = 6) were immunized with 2 × 10^10^ vp of AdC68-S or AdC6-S (day 0), and the prime-boost groups were boosted with the same dose of heterologous chimpanzee adenoviral vaccine (day 0 and day 30) via the i.m. route. Mice of control groups were vaccinated with 2 × 10^10^ vp of single-dose AdC6-empty and AdC68-empty (day 0), respectively. Blood samples were collected for antibody titration before SARS-CoV-2 challenge. Mice were i.n. infected with 8 × 10^9^ vp of Ad5-hACE2 at day 57 after initial immunization and challenged with 5 × 10^5^ TCID_50_ SARS-CoV-2 via the i.n. route at five days post transduction. Three days post challenge, mice were euthanized, and lung tissues were harvested for virus titration and pathological examination. **b** Serum samples were collected before challenge (day 56) and live virus-neutralizing antibodies NT_50_ titers were assessed. **c** SARS-CoV-2 viral titers of lung tissues were measured using RT-PCR probing virus genome RNA. Dotted lines indicate the LLOD. Values below the LLOD were set to half of the LLOD. **d** Copies of SARS-CoV-2 viral sgRNA in lungs. **e** Tissue sections of the lung tissues of six mice per group were stained with H&E for pathological examination, and representative photomicrographs of both low magnifications (scale bars, 200 μm) and high magnifications (scale bars, 50 μm) from each group are shown. All data were shown as means ± SEM. *P*-values were analyzed with one-way ANOVA (^ns^*P* ≥ 0.05; *P < 0.05; **P < 0.01; ***P < 0.001; ****P < 0.0001).

A rapidly generated mouse model^15^ was used to test the protective efficacy of vaccine candidates. Six 6–8-week-old female BALB/c mice per group received vaccines using a prime-only regimen (day 0) or prime-boost regimen (day 0 and day 30), with 2 × 10^10^ vp via the i.m. route. As controls, six animals per group were vaccinated using a prime-only regimen with the same dose of AdC6-empty and AdC68-empty. Subsequently, the recombinant Ad5 expressing hACE2 was transduced into mouse lungs via the intranasal (i.n.) route on day 57 after the initial immunization. Five days later, all mice were challenged with 5 × 10^5^ TCID_50_ (50% tissue culture infective doses) live SARS-CoV-2. Sera were collected for antibody detection before the challenge, and lungs of mice were harvested for viral load detection and pathologic analysis on day 3 post-challenge (Fig. 3a). High endpoint titers of spike-specific binding antibodies and pseudovirus neutralizing antibodies were detected at week 2, and levels stably increased at week 4 after the initial vaccination (Supplementary Fig. S4a, b). After the boosting immunization, the antibody titers in mice immunized with AdC68-S + AdC6-S prime-boost vaccine increased rapidly compared with a slight increase in other groups at week 6, and the average binding antibody titers and pseudovirus neutralizing antibody titers reached the highest levels of 23,979 and 515 at week 8, respectively (Supplementary Fig. S4a, b). Neutralizing antibodies against live virus SARS-CoV-2 were detected before challenge, and virus-specific neutralizing antibodies were detected in all vaccinated mice. The GMT of AdC6-S and AdC68-S prime-only groups were 508 and 180, the AdC6-S + AdC68-S and AdC68-S + AdC6-S prime-boost group were 320, and 403, respectively (Fig. 3b). Titers of pseudovirus neutralizing antibodies correlated with both anti-spike total IgG titers and live virus-neutralizing antibody titers (*R* = 0.9872, *P* < 0.0001 and *R* = 0.9627, *P* < 0.0001, respectively) (Supplementary Fig. S4c, d). IgG antibody isotype profiles in vaccinated BALB/c mice were also measured, and high endpoint titers of IgG1, IgG2a, and IgG2b were produced in all immunized mice, and no detectable antibodies were detected in the control groups (Supplementary Fig. S5a–f). In addition, the IgG2a/IgG1 ratios in all vaccinated groups were ~1.0, presenting a balanced Th1/Th2 response (Supplementary Fig. S5g, h).

Viral genomic RNA (gRNA) was measured using quantitative real-time reverse-transcription PCR (RT-PCR) in mouse lung tissues after the challenge. The mean titers of viral gRNA (log_10_) for control groups were 7.45 (AdC6-empty) and 7.12 (AdC68-empty) copies/g. For mice immunized with AdC6-S, AdC68-S, AdC6-S + AdC68-S and AdC68-S + AdC6-S, the mean titers of gRNA (log_10_) were 5.44, 4.44, 4.7 and 3.65 copies/g, respectively (Fig. 3c). Strikingly, the titers of gRNA (log_10_) in AdC68-S + AdC6-S group were all below the lower limit of detection (LLOD, 3.95), showing a complete protection against live SARS-CoV-2 challenge. In addition, viral gRNA copies in the lungs were negatively correlated with live virus-neutralizing antibody titers in vaccinated mice (*R* = –0.7139, *P* < 0.0001; Supplementary Fig. S4e). Subgenomic RNA (sgRNA), which is indicative of virus replication, was then detected by using RT-PCR. The mean titers of viral sgRNA (log_10_) for control groups were 8.08 (AdC6-empty) and 6.84 (AdC68-empty) copies/g. For mice immunized with AdC6-S, AdC68-S, AdC6-S + AdC68-S and AdC68-S + AdC6-S, the mean titers of sgRNA (log_10_) were all 4.88 copies/g, below the LLOD (5.18) (Fig. 3d). These results suggest no viral replication in all vaccinated mice. Thereafter, sections of mouse lung tissues were fixed and stained with hematoxylin and eosin (H&E), and pathological observations were assessed 3 days post challenge. Lung tissues of mice from the two control groups (AdC6-empty or AdC68-empty alone) showed typical viral interstitial pneumonia, a key feature of COVID-19, characterized by thickened alveolar walls and heavy infiltration of mononuclear inflammatory cells into the alveolar interstitium, accumulation of macrophages in alveolar cavities, hemorrhage, and disappearance of recognizable architectures. However, no visible pathological damage or inflammatory response was observed in lung tissues from mice vaccinated with vaccine candidates (Fig. 3e), and pathogenic changes were scored to evaluate lung damage (Supplementary Fig. S4f).

### Single-cell transcriptional landscape of splenocytes from vaccinated mice

To investigate the immunological effect and underlying mechanism of action of adenovirus-based COVID-19 vaccine, 6–8-week-old female C57BL/6 mice were vaccinated, and one animal per group was immunized with a single dose of AdC6-S or AdC68-S vaccine candidate, or prime-boost vaccine (AdC68-S + AdC6-S), respectively, with 2 × 10^10^ vp via the intramuscular route. As controls, mice received the same doses of AdC6-empty and AdC68-empty using the same regimens. One C57BL/6 naive mouse was used as a blank control. The splenocytes of each animal from all groups were isolated at week 7 after initial immunization, and droplet-based scRNA-seq analysis was performed on the 10× genomics platform (Supplementary Fig. S6a). After filtering out low-quality cells, a total of 40,497 cells in all samples were obtained, and principal component analysis (PCA) was performed by integrating high-quality cells into an unbatched and comparable dataset. The transcriptomes of the eight main cell types of immunocytes were captured using graph-based clustering of uniform manifold approximation and projection (UMAP) (Supplementary Fig. S6b), and immunocytes including T cells, B cells, monocytes, macrophages, NKT, DCs, NK, and granulocytes, were identified based on the expression of cell-type markers (Supplementary Fig. S6c). The proportions of each cell type in naive mice, mice injected with empty adenovirus particles, and mice immunized with vaccine candidates using different regimens were compared. We found that T cells and B cells were the major components of captured splenic immunocytes, and that their proportions were significantly higher in mice injected with adenovirus particles than that in naive mice. In contrast, except for a mild decrease in NK cells and macrophages, a slight increase in granulocytes and no other changes in cell subtypes were found in vaccinated mice (Supplementary Fig. S6d).

**Fig. 4:**
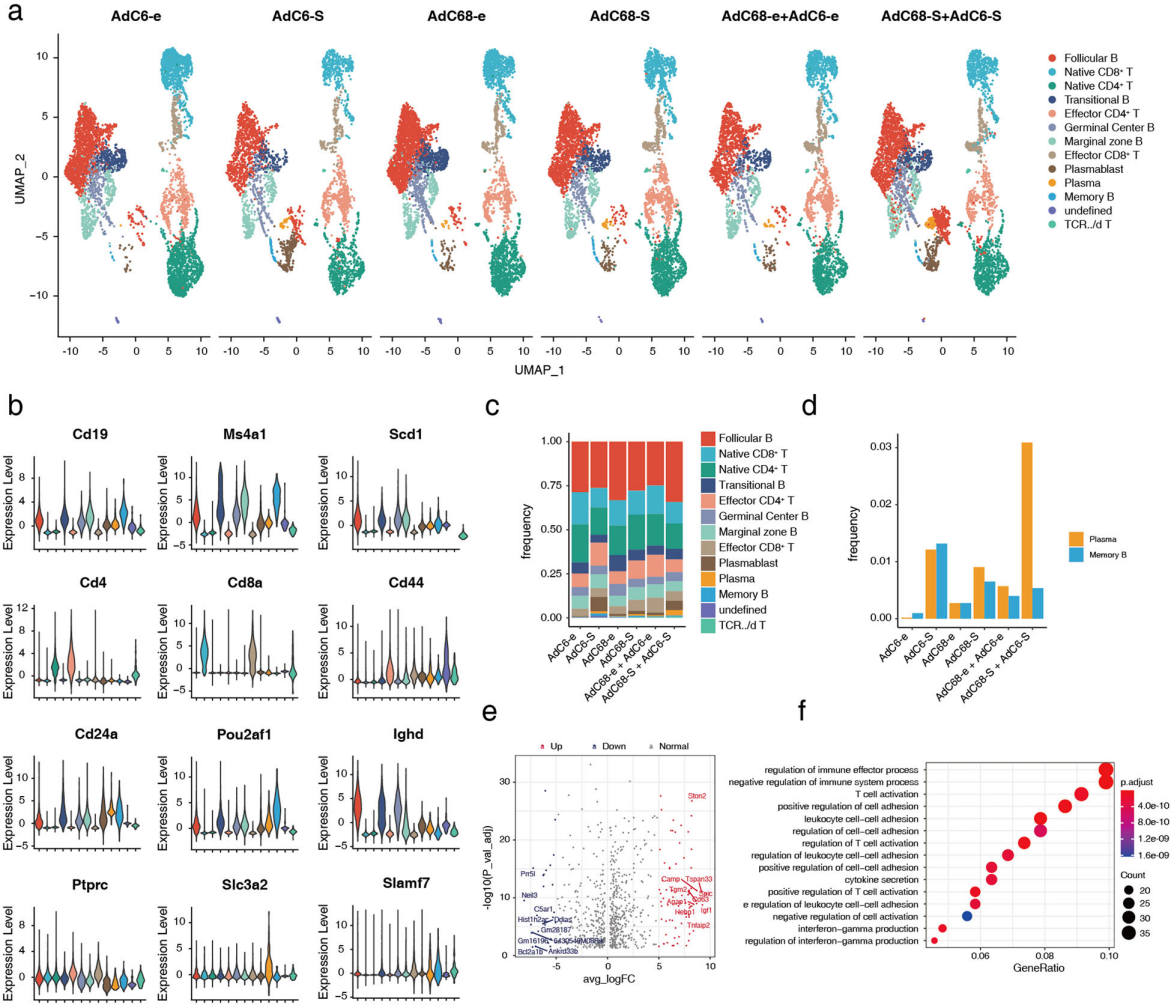
Heterologous prime-boost vaccination generates more plasma cells with superior humoral immune responses in C57BL/6 mice. **a** UMAP dimensionality reduction embedding of T cells and B cells from vaccinated C57BL/6 mice. Cells are colored according to lineage subtypes. **b** Volcano plot displaying the expression levels of cell typing genes in subtype clusters. **c** Barplot of lineage subtype frequency in each group. **d** Bar graph summarizing the frequencies of plasma cells and memory B cells in each sample. **e** Volcano plot of DEGs in plasma cells and memory B cells of the group of mice vaccinated with AdC68-S and boosted with AdC6-S, compared to groups of mice immunized with AdC68-S and AdC6-S via single-dose route, respectively. Red dots represent genes significantly upregulated in prime-boost group (adjusted *P* < 0.05 and FC (fold change) ≥ 5), while blue dots represent significantly downregulated genes (adjusted *P* < 0.05 and FC ≤ 5). Top 10 genes are labeled by gene symbols. **f** Top 15 enriched GO terms for upregulated genes in prime-boost group compared to those in AdC68-S and AdC6-S single-dose groups in plasma cells and memory B cells. Dot color indicates the statistical significance of enrichment and dot size represents gene ratio annotated to each term.

We further analyzed the drastic changes in T cells and B cells in the vaccinated group and obtained 12 subclusters according to the expression of marker genes of each cell type (Fig. 4a, b). The frequencies of cell components were demonstrated as histograms, and follicular B cells were easily identified as the major component among all cell types in all vaccinated groups (Fig. 4c). The induction of strong and long-term neutralizing antibodies is key for successful vaccination, and most efficient vaccines generate prolonged immune responses and consequent immunological memory by eliciting long-lived plasma cells and memory B cells^17^. Therefore, we evaluated the frequencies of plasma cells and memory B cells in the immunized mice. More plasma cells and memory B cells were generated in mice administered vaccine candidates compared with those in mice injected with adenoviral empty vectors; however, mice vaccinated using the prime-boost strategy elicited more plasma cells than those with the single-dose vaccination at week 7; more memory B cells were generated in mice with single-dose immunization, especially in mice vaccinated with the AdC6-S vaccine candidate (Fig. 4d). We further explored the differential gene expression between the prime-boost group (AdC68-S + AdC6-S, *n* = 1) and single-dose groups (AdC6-S and AdC68-S, *n* = 2). Compared with the single-dose groups, the number of upregulated genes was higher than that of downregulated genes in the prime-boost group, and the top five genes were *Spic*, *Igf1*, *Tspan33*, *Camp*, and *Cd63* (Fig. 4e). In addition, Gene Ontology (GO) analyses were performed to gain insight into the biological functions of the upregulated genes. The upregulated genes that were most significantly enriched were associated with cell–cell adhesion, T cell activation, and immune effector processes (Fig. 4f).

### Vaccines using prime-boost regimen trigger a robust germinal center (GC) response

To further investigate the difference between prime-boost group and prime-only groups, the differential gene expression in T cells was first analyzed. Interestingly, we found that *Icos* (encoded ICOS) and *Cd40lg* (encoded CD40L) genes were highly expressed in effector CD4^+^ T subcluster cells of AdC68-S + AdC6-S group, compared with AdC6-S and AdC68-S prime-only groups (Supplementary Fig. S6e, f). ICOS is homologous to CD28 but is expressed on T cells only after activation^18^. ICOS is actively involved in promoting GC formation and effective interaction between T and B cells^19^. We then found that *Cd40* gene, which encodes CD40, a binding partner of CD40L, was also highly expressed in follicular B cells in prime-boost group (Supplementary Fig. S6g). The interaction between B cell-expressed CD40 and CD40L, which is predominantly expressed on activated CD4^+^ T cells, plays a critical role in promoting GC formation^20^. This indicated that CD4^+^ T were activated and are likely to participate in T cell-dependent B cell responses in spleen of AdC68-S + AdC6-S prime-boost group.

**Fig. 5:**
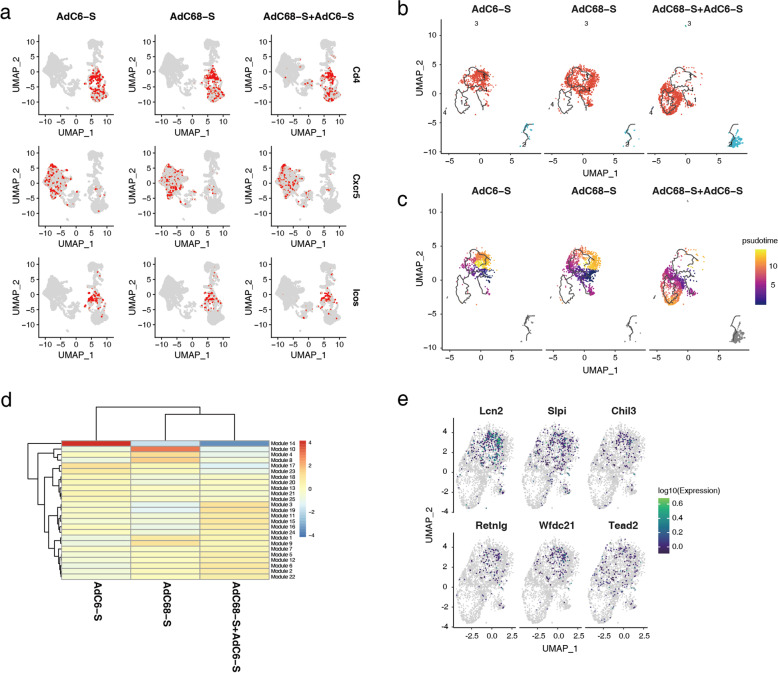
Heterologous prime-boost strategy of vaccine candidates promoting differentiation of follicular B cells through scRNA-seq analysis. **a** UMAP of Tfh cells in each vaccinated group, and expression levels of representative marker genes of Tfh cells are plotted. The color key from gray to red indicates relative expression levels from low to high, respectively. **b** Reconstruction of the developmental trajectory of follicular B cells using the Monocle 3 algorithm, and four subgroups were identified. Each cell is colored by its subgroup category and displayed on UMAP. **c** UMAP of single-cell pseudotime trajectory of follicular B cells; cells are ordered in pseudotime and colored in a gradient from purple to yellow. **d** Heatmap displaying modules of co-regulated genes in subgroup 1 for follicular B cells. **e** UMAP of DEGs in module 10 and module 14.

During T cell-dependent B cell responses, the formation of GCs is the anatomical hallmark of T helper cell activity and the physical site of T cell/B cell interaction^19^. Naive B cells encounter and internalize the antigen, then move to the T/B cell border, with the help of T follicular helper (Tfh) cells, antigen-specific B cell proliferate to form GC and then develop into plasma cells or memory B cells^21^. To further confirm whether immune responses of different vaccination regimens correlated with GC responses, Tfh cells were measured using signature marker genes, such as *CD4*, *CXCR5*, and *ICOS* (Fig. 5a). Mice immunized with the AdC68-S + AdC6-S prime-boost regimen and a single dose of AdC6-S or AdC68-S all elicited mild Tfh responses after seven weeks of initial immunization, and no significant difference in the numbers of Tfh cells between these groups. Follicular B cells are a mature B lymphocyte population that recirculates between lymphoid organs, and key cells to form GC. Consequently, we reconstructed the developmental trajectory of follicular B cells (Fig. 5b, c), the largest proportion of B cells in vaccinated mice (Fig. 4c). Cells were organized based on gene expression using an unsupervised inference analysis (Monocle 3); four supergroups were visualized via projection for dimension reduction (UMAP) algorithm, and supergroup 1 was changed significantly in mice immunized with three vaccine candidates (Fig. 5b). To investigate further details in supergroup 1, pseudotime analysis was performed (Fig. 5c). Interestingly, similar branching trajectories were observed in follicular B cells in mice vaccinated with single-dose AdC6-S or AdC68-S, but differentiated from the AdC68-S + AdC6-S prime-boost group. To indicate how gene expression profiles differed among the three groups, a heatmap was generated to arrange the genes of supergroup 1 into modules with common expression profiles, and modules 14 and 10 were found to be significantly downregulated in the prime-boost group (Fig. 5d). Furthermore, we used monocle 3 to fit a regression model to each gene in modules 14 and 10, and further identified the differentially expressed genes (DEGs) in the three groups (Fig. 5e), including *Lcn2*, *Slpi*, *Chil3*, *Retnlg*, *Wfdc21*, and *Tead2*, which were overlaid on the UMAP of single-cell data.

## Discussion

Since the SARS-CoV-2 worldwide outbreak, several vaccines for preventing COVID-19 have been authorized for emergency use worldwide. In spite of this, the global development and deployment of vaccines is still of critical priority^22^. Replication-deficient recombinant chimpanzee adenoviral vector has been proven to be safe, tolerable, and efficient at inducing potent and sustained specific T and B cell responses and good protective efficacy in previous clinical studies^23–25^. To date, at least four COVID-19 vaccines have been based on an adenoviral vector strategy including Ad5^4,26^, Ad26^5^, ChAdOx1^6,25,27^, and a combination of Ad5 and Ad26^28^. Among these, Ad5 has already been widely manipulated in vaccine development, although Ad5 has a high seroprevalence in humans, which may weaken vaccine efficacy, and high-dose administration may contribute to a high risk of side effects^29^. AdC68 and AdC6, the two adenoviral vectors derived from a chimpanzee adenovirus, can overcome the shortcomings of pre-existing immunity, because they have an extremely low seropositivity compared to that of Ad5^29,30^, or even compared to that of Ad26, a rare human adenovirus serotype^31^. At present, AZD1222 is a chimpanzee adenoviral-vectored vaccine developed in clinical trials, which uses ChAdOx1 as the vaccine vector and has already been administered to the population. A previous study showed that AdC68 can induce immune responses comparable to those induced by ChAdOx1^32^. By combination with AdC6, the effectiveness of vaccines may be greatly improved. Additionally, except for one human clinical study performed in Russia that combined two different vectors, Ad5 and Ad26^7,28^, most of these clinical studies were based on two or multiple doses of the same component. The preliminary results of the Russia prime-boost vaccine showed 91.6% efficacy and safety insurance in the phase 3 clinical trial^28^. This showed the highest protection efficacy among the SARS-CoV-2 vaccine studies based on adenoviral vectors, even comparable to the results of mRNA vaccines developed by Moderna and Pfizer/BioNTech, with protective efficacies of 94.1% and 95%, respectively^33,34^. By combining heterologous adenoviral vectors, the influence of the negative immune response induced by the vector itself can be ignored. In most cases, a heterologous prime-boost approach is more effective than homologous prime-boost approach^12,35^.

In our study, AdC68-S + AdC6-S combination showed better immune responses than AdC6-S + AdC68-S. The immune effect of prime-boost regiment is related to the vaccine candidates, doses, and pre-existing immunity against the vectors themselves^35^. Firstly, vector-specific neutralizing antibodies can affect the prime-boost efficacy. In order to exclude the possible cross-reaction between the two vector-specific nAbs, we tested nAbs against AdC6 and AdC68 vectors in serum samples of C57BL/6 mice injected with corresponding vaccine candidates. No cross-reaction between these two nAbs was observed, suggesting that nAbs against these two vectors do not account for the different immune effect between groups. Notably, we found that AdC6-S and AdC6-empty adenovirus induced high nAbs against AdC6 vectors, while AdC68 adenovirus only elicited anti-vector antibody responses in homologous prime-boost strategies (Supplementary Fig. S8a–f). Secondly, to further explore the immunogenicity of the vectors, the infectious units were titrated on HEK293 cells by hexon staining. The infectious titer of AdC6-S is 4.28 × 10^10^ ifu/mL, and AdC68-S is 2.01 × 10^9^ ifu/mL. In our study, each adenovirus was used at 2 × 10^10^ vp for immunization, which was measured by ultraviolet spectrophotometer. Therefore, a lower dose of AdC68-S (1 × 10^7^ ifu) at priming, followed by a higher dose of AdC6-S (1 × 10^8^ ifu) at boosting, elicited more effective immune responses. Therefore, we suspect that the difference of infectious units between these two adenoviruses is the main reason why AdC68-S + AdC6-S prime-boost group is better. Interestingly, studies have shown that lower doses of antigen at priming greatly induce the immune memory, whereas higher doses generally stimulate the effector cells. In comparison with the prime dose, higher dose of the booster could induce higher levels of immune response^35,36^. This might explain why AdC68-S + AdC6-S prime-boost group showed the best immune effect in our study. More prime-boost groups at different dose combinations should be included to further validate this hypothesis. We reduce the immune dose to further explore the immune profiles of different prime-boost vaccination of AdC6-S and AdC68-S (Supplementary Figs. S7 and S8). Adenoviruses were re-amplified and purified, and the infectious units were titrated. Then, low dose (1 × 10^8^ vp), middle dose (1 × 10^9^ vp), high dose (1 × 10^10^ vp) was administrated via intramuscular injections, respectively. Heterologous prime-boost elicited higher spike-specific binding antibodies and SARS-CoV-2 pseudovirus-based neutralizing antibodies than homologous prime-boost and single-dose immunization (Supplementary Fig. S7).

Vaccine-associated enhanced respiratory disease (VAERD) is a distinct clinical syndrome that occurs in young children immunized with whole-inactivated virus vaccines against measles and respiratory syncytial virus (RSV)^37,38^, and VAERD should also be considered for COVID-19 vaccines, because a similar performance was observed in animals administered whole-inactivated SARS-CoV vaccine^39,40^. The induction of non-neutralizing antibodies and Th2-based immune responses is associated with VAERD. In this study, a high level of neutralizing antibody was elicited, balanced Th1/Th2 immune responses were induced through S-specific IgG subtype profile tests, and no Th2 cells could be detected, regardless of whether the administration was single-dose or prime-boost vaccination, suggesting that our two vaccine candidates avoid Th2-based immune response and the occurrence of VAERD.

Herein, we first exploited two heterologous chimpanzee Ad vectors as prime-boost vaccines against SARS-CoV-2 and found the most efficient strategy of AdC68 priming with AdC6 boost. These findings indicate that our heterologous prime-boost vaccine may serve as a promising vaccine candidate against SARS-CoV-2 and provide a novel strategy for clinical use.

## Materials and methods

### Animals and ethics statement

For immunization experiments, 6–8-week-old female C57BL/6 mice were purchased from SPF Biotechnology Co., Ltd. (Beijing, China) and housed in specific pathogen-free (SPF) animal facilities at Tianjin Medical University, Tianjin, China. All the animal procedures were approved by the Committee on the Use and Care of Animals of Tianjin Medical University. For the challenge experiment, 6–8-week-old female BALB/c mice were purchased from Beijing Vital River Laboratory (Beijing, China) and housed under SPF conditions in the laboratory of animal facilities at the Institute of Microbiology, Chinese Academy of Science (IMCAS), Beijing, China. And animal experiments were approved by the Committee on the Ethics of Animal Experiments of the IMCAS, and conducted in compliance with the recommendations in the Guide for the Care and Use of Laboratory Animals of the IMCSA Ethics Committee. While those related to the live SARS-CoV-2 virus were conducted at the Animal Biosafety Level 3 (ABSL3) facility in IMCAS following the standard operating protocols approved by the Committee on the Ethics of Animal Experiments of the IMCAS. We have complied with all relevant ethical regulations for animal testing and research.

### Cells and viruses

HEK293A, HEK293T, Huh7, VERO-E6 were purchased from the American Type Culture Collection (ATCC), and HEK293T cells overexpressing human ACE2 were provided by Yang Liu (Institute of Radiation Medicine, Chinese Academy of Medical Sciences and Peking Union Medical College), BHK cells overexpressing human ACE2 were provided by Xin Zhao (Institute of Microbiology, CAS). Cells were maintained in complete Dulbecco’s modified Eagle’s medium (DMEM, HyClone), supplemented with 10% (v/v) fetal bovine serum (FBS), 100 U/mL penicillin, and 100 µg/mL streptomycin, and incubated at 37 °C under 5% CO_2_. SARS-CoV-2 (hCoV-19/China/CAS-B001/2020, GISAID No. EPI_ISL_514256-7) was propagated in VERO-E6 cells and titrated by TCID_50_ assay on VERO-E6^41^. Recombinant type 5 adenovirus expressing human ACE2 protein was provided by G. Wong (Institut Pasteur of Shanghai, CAS).

### Vaccine generation

Recombinant adenoviral AdC6 and AdC68 vectors were generated via one-step isothermal assembly as previously described^42^. AdC6 and AdC68 vectors incorporating the full-length spike gene of the SARS-CoV-2 strain Wuhan-Hu-1 (GenBank accession no. NC_045512) were constructed. Briefly, spike genes were codon-optimized and synthesized by TSINGKE (Beijing, China), and inserted into the E1-deleted region of the replication-deficient adenoviral vectors AdC6 and AdC68, respectively, driven by a modified human cytomegalovirus major immediate early promoter (IE CMV). AdC6-empty and AdC68-empty were employed as sham controls, with no insertion at the E1-deleted locus. Recombinant adenoviruses were rescued and propagated in HEK293A cells and purified by CsCl gradient ultracentrifugation, as previously described^14^, and viral particles were calculated using spectrophotometry, and the infectious units were titrated on HEK293A cells using Quick Titer^TM^ Adenovirus Titer Immunoassay Kit (Cell Biolabs) following the manufacturer’s instructions. Adenovirus vectors were sequenced and identified by restriction enzyme digestion.

### Western blot assay

HEK293A cells were cultured and seeded on 6-well plates before transfection, and then infected with either AdC68-S or AdC6-S at varying doses of 1 × 10^8^, 1 × 10^9^, and 1 × 10^10^ vp each well, and 1 × 10^10^ vp of AdC68-empty and AdC6-empty were used as controls. Cells were collected and lysed in 200 μL RIPA buffer containing protease inhibitors (Roche) 24 h post-infection. Each cell lysate was subjected to 8% SDS-polyacrylamide gel electrophoresis (SDS-PAGE), transferred to polyvinylidene difluoride (PVDF) membrane, blotted with anti-SARS-CoV-2 Spike RBD polyclonal antibody (SinoBiological), and goat anti-rabbit IgG H&L (horseradish peroxidase (HRP)-conjugated) (Abcam). HRP-α-tubulin mouse polyclonal antibody (Proteintech) was used as an internal control. All blots derived from the same experiment and were processed in parallel.

### Vaccinations of mice and subsequent virus challenge

6–8-week-old female C57BL/6 mice were randomly divided into eight groups and administered 2 × 10^10^ vp vaccine candidates via the i.m. route, using prime-only regimens (AdC6-S or AdC68-S, day 0) or prime-boost regimens (AdC6-S + AdC68-S or AdC68-S + AdC6-S, day 0 and day 28). Mice in the control groups were immunized with empty adenovirus vectors using the same regimens. Sera were collected to detect antibody titers (*n* = 5), and splenocytes were harvested to assess the T cell response (*n* = 6).

Six female BALB/c mice at 6–8 weeks of age per group were immunized with 2 × 10^10^ vp of vaccine candidates via the i.m. route using prime-only regimens (AdC6-S or AdC68-S, respectively, day 0) or prime-boost regimens (AdC6-S + AdC68-S or AdC68-S + AdC6-S, respectively, day 0 and day 30), and serum samples were collected before the challenge. To evaluate the protective efficacy of vaccine candidates against live SARS-CoV-2, a recombinant human adenovirus serotype 5 expressing human ACE2 (Ad5-hACE2)-transduced BALB/c mice model was used^15^. Immunized BALB/c mice were i.n. infected with 8 × 10^9^ vp of Ad5-hACE2 at day 57 and i.n. challenged with 5 × 10^5^ TCID_50_ of live SARS-CoV-2 (hCoV-19/China/CAS-B001/2020, GISAID No. EPI_ISL_514256-7) 5 days later. Three days post-challenge, mice were euthanized and necropsied. Lung tissues were collected and split into two parts for virus titration and pathological examination. All animal experiments with SARS-CoV-2 challenge were conducted at the Animal Biosafety Level 3 (ABSL3) facility in IMCAS.

### ELISA

Spike-specific binding antibodies were assessed by ELISA, as previously described^12^. Briefly, 100 ng of spike proteins (SinoBiological) in Na_2_CO_3_-NaHCO_3_ buffer (pH 9.6) were coated onto a 96-well ELISA plate (Corning) and incubated at 4 °C overnight. After incubation, plates were washed three times with PBST (0.05% Tween-20 in 1× DPBS) and blocked with 200 μL of 5% skim milk per well, at 37 °C for 2 h. Three-fold serial dilutions of heat-inactivated serum in 1% BSA, at a starting dilution of 1:400, were added to the wells and incubated at 37 °C for another 2 h, while the sera of the control groups were diluted at a starting dilution of 1:100. Plates were washed three times with PBST and then incubated with 1:100,000 dilutions of HRP-conjugated anti-mouse IgG (Abcam), or 1:5000 dilutions of HRP-conjugated anti-mouse IgG2a, IgG2b, or IgG1 (Southern Biotechnology) for 1 h at 37 °C. Plates were washed three times and then 50 μL of 3,3′,5,5′-tetramethylbenzidine (TMB) substrate (Thermo Fisher) was added to each well, and 2 M sulfuric acid (H_2_SO_4_) solution of equal volume was used to stop the color reaction. The absorbance at 450 and 630 nm was recorded using a microplate reader (Bio Tek). The binding antibody endpoint titer was determined as the reciprocal of the highest serum dilution that yielded an absorbance greater or equal to 0.1 OD unit above the absorbance of the pre-immune samples.

### Production and titration of SARS-CoV-2 pseudoviruses

The codon-optimized gene encoding SARS-CoV-2 spike protein with and without C-terminal 19-amino acid deletion of Wuhan-Hu-1 strain (YP 009724390.1) was synthesized by TSINGKE (Beijing, China), and cloned into pcDNA3.1(+) vector (pS-CΔ19-Wuhan, pS-Wuhan, respectively). pS-B.1.1.7 and pS-B.1.351 plasmids were constructed with mutant S genes expressing the spike protein of the B.1.1.7 variant (GenBank: QQH18545.1, containing the H69, V70, and Y145 deletions and N501Y, A570D, D614G, P681H, T716I, S982A, and D1118H mutations) and B.1.351 variant (GenBank: QRI43207.1, containing the L242, A243, and L244 deletions and L18F, D80A, D215G, S305T, K417N, E484K, N501Y, D614G, and A701V mutations) using the same method. The SARS-CoV-2 spike-based pseudoviruses expressing the firefly luciferase reporter gene were generated as previously described^43,44^. Briefly, SARS-CoV-2 pseudoviruses were produced using HEK293T cells co-transfected with polyethyleneimine (Polysciences) using pNL4-3.Luc.R-E- backbone plasmid and expressing plasmid pS-CΔ19-Wuhan, pS-Wuhan, pS-B.1.1.7, and pS-B.1.351, respectively, at a 15:1 ratio. Forty-eight hours post-transfection, supernatants were collected and filtered with 0.45 μm filters, concentrated overnight with 5% polyethylene glycol 8000 at 4 °C, centrifuged for 30 min at 5000× *g*, resuspended, and stored at –80 °C. Viral RNA was extracted using TRIzol (Sigma), and the number of viral RNA genomes per mL of viral stock solution were determined using RT-qPCR with the sense primer: 5′-TGTGTGCCCGTCTGTTGTGT-3′, and anti-sense primer: 5′-GAGTCCTGCGTCGAGAGAGC-3′. The known quantity of the pNL4-3 Luc.R-E-plasmid was used to generate standard curves, and the titers of pseudoviruses were calculated. Pseudotyped viruses with spikes in the Wuhan-Hu-1 strain, B.1.1.7, and B.1.351 variants were adjusted to the same titer for the neutralization assay.

### Pseudovirus neutralization assay

To determine the neutralization activity of the sera from vaccinated mice against SARS-CoV-2, Huh7 cells were pre-seeded in 96-well tissue culture plates at a density of 2.5 × 10^4^ cells per well. Serially diluted heat-inactivated serum samples at a starting dilution of 1:20 were prepared and mixed with an equal volume of a fixed amount of SARS-CoV-2 pseudoviruses bearing spike protein with C-terminal 19-amino acid deletion of Wuhan-Hu-1 strain, and incubated at 37 °C for 2 h; the mixtures were then added back to the pre-seeded Huh7 cells. Moreover, to assess the titers of pseudovirus neutralizing antibodies against SARS-CoV-2 original Wuhan-Hu-1 strain, B.1.1.7, and B.1.351 variants in serum samples of vaccinated mice simultaneously, 50 μL pseudoviruses, equivalent to 3.8 × 10^4^ vector genomes, were incubated with an equal volume of inactivated sera with serial dilutions at 37 °C for 1 h. Samples were then added to the HEK293T-hACE2 cells (with two replicates for each dilution), which were pre-seeded on 96-well plates (2.5 × 10^4^ cells per well). Forty-eight hours post incubation, cells were lysed and relative luciferase activity (RLA) was measured using the Steady-Glo^®^ Luciferase Assay System (Promega). NT_50_ was calculated as the serum dilution at which RLA was reduced by 50% compared with RLA in virus control wells.

### Live SARS-CoV-2 neutralization assay

SARS-CoV-2 (hCoV-19/China/CAS-B001/2020, GISAID No. EPI_ISL_514256-7) was propagated in VERO-E6 cells and titrated by TCID_50_ assay on VERO-E6. The sera of immunized mice were serially diluted four-fold, mixed with the same volume of SARS-CoV-2 (100× TCID_50_), and incubated at 37 °C for 1 h. Thereafter, 100 μL of the virus-serum mixture was transferred to pre-plated BHK-hACE2 cells in 96-well plates. Inoculated plates were incubated at 37 °C for an additional 72 h, following which the cytopathic effect was observed microscopically. The neutralization titers were defined as the reciprocal of the serum dilution required for 50% neutralization of viral infection. All live virus neutralization assays were conducted in the biosafety level 3 (BSL3) facility of IMCAS.

### Adenovirus neutralization assays

An adenovirus neutralization assay was performed based on previously described methods^45^. Before testing, an optimal virus concentration to use in neutralization should be determined. Briefly, 50 μL of AdC6-eGFP or AdC68-eGFP adenoviruses expressing green fluorescent protein were added to 96-well plate with 10-fold serial dilution by DMEM with 5% FBS, followed by 50 μL medium, and 100 μL of HEK293A cell suspension (2.5 × 10^5^ cells/mL) was added subsequently. Plates were analyzed and photographed using a fluorescence microscope (Olympus) after incubation at 37 °C in a 5% CO_2_ atmosphere for 24 h. 50 μL of the immunized mice sera were serially diluted two-fold (1:20 to 1:40,960), mixed with the same volume of AdC6-eGFP or AdC68-eGFP, and incubated at 37 °C for 1 h, cell suspension was then added per well. DMEM without serum was used as the negative control. The neutralizing antibody titer was expressed as the reciprocal of dilutions for which the proportion of GFP-expressing cells was reduced to ~50% of that for the negative control. A titer ≥ 20 was regarded as positive for the serotype-specific neutralizing antibodies.

### Peptides and stimulation

A total of 316 peptides spanning the full-length spike glycoprotein of SARS-CoV-2 were synthesized as 15-mers, with 11 overlapping amino acids for antigen-specific T cell assay (GenScript, RP30020). Peptides were delivered into two peptide pools, representing the S1 subunit (158 peptides scanning 1–643 aa of spike) and the S2 subunit (other peptides spanning 644–1273 aa of spike), respectively. The two peptide pools were used separately for flow cytometry and ELISpot assays.

### Flow cytometry

T cell responses on day 10 post priming and boosting immunization of vaccines were evaluated using intracellular cytokine staining, and a single-cell suspension of murine splenocytes was prepared by passing cells through 70 μm strainers and ACK lysis prior to resuspension in complete RPMI medium supplemented with 10% (v/v) FBS, 100 U/mL penicillin, and 100 µg/mL streptomycin. 1 × 10^6^ cells were plated in 96-well tissue culture plates and stimulated with two separate peptide pools, at a final concentration of 1 μg/mL for 4 h, and subsequently cultured for 8 h with Golgi-Plugs (BD Bioscience) at 37 °C and 5% CO_2_. Cell stimulation cocktails (Invitrogen) and complete RPMI medium were used as positive and negative controls, respectively. Cells were then washed with MACS buffer (phosphate-buffered saline with 0.5% bovine serum albumin and 5 mM EDTA-Na2), stained with LIVE/DEAD Fixable Aqua Dead Cell Stain Kit (Invitrogen) and anti-CD45-APC-Cy7, anti-CD3e-PerCP-Cy5.5, anti-CD4-FITC, anti-CD8-PE-Cy7 (BD Bioscience), and anti-CD44-PE-eFluor610 (eBioscience) surface antibodies for 30 min at 4 °C. Cells were then resuspended in Fixation/Permeabilization solution (BD Bioscience) for 20 min at 4 °C, washed in Perm/Wash buffer, and stained with intracellular antibodies, including anti-IL-2-PE (eBioscience), anti-IFN-γ-APC (BD Bioscience), and anti-TNF-α-BV421 (BD Bioscience) at 4 °C for 30 min. Cells were fixed with 2% paraformaldehyde for 30 min at 4 °C, washed twice in MACS buffer, resuspended in 300 μL of PBS buffer for acquisition using BD LSRFortessa (BD Bioscience) and analyzed using FlowJo v10.

### IFN-γ ELISpot assays

ELISpot assays were performed on isolated murine splenocytes. ELISpot plates (Millipore) were pre-wetted by adding 50 μL 70% ethanol per well for 2 min, washed five times with sterile water, and pre-coated overnight with capture antibody AN18 (Mabtech), and diluted in sterile PBS (15 μg/mL) at 4 °C. The plates were blocked with complete RPMI medium for at least 30 min at room temperature before seeding 5 × 10^4^ splenocytes into each well, and separately stimulated with the S1 and S2 peptide pools, as described above, for 48 h in a 37 °C and 5% CO2 incubator; cell stimulation cocktails (Invitrogen) and complete RPMI medium were used as positive and negative controls. IFN-γ spot forming units (SFU) were detected by staining PVDF membranes with detection antibody R4-6A2-biotin (Mabtech) at 1 μg/mL for 2 h at room temperature, followed by streptavidin-horseradish peroxidase diluted (1:1000) in PBS containing 0.5% FBS for 1 h at room temperature, and developed with TMB substrate solution (Mabtech). Spots were scanned and quantified using an AT-Spot reader (China), and SFU per million cells was calculated.

### RT-PCR assay

Mouse lung tissues were weighed and homogenized. Virus RNA was isolated from 50 μL supernatants of homogenized tissues using a MagMAX™ Express Magnetic Particle Processor nucleic acid extraction instrument (Applied Biosystems). SARS-CoV-2-specific RT-PCR assays were performed using a FastKing One Step Probe RT-qPCR kit (Tiangen Biotech) on a CFX96 Touch Real-Time PCR Detection System (Bio-Rad) according to the manufacturer’s protocol. Two sets of primers and probes were used to detect a region of the N gene of viral genome and a region of E gene of sgRNA of SARS-CoV-2, respectively^41^. The primer and probe sequences were as follows: N-F, GACCCCAAAATCAGCGAAAT; N-R, TCTGGTTACTGCCAGTTGAATCTG; N-probe, FAM-ACCCCGCATTACGTTTGGTGGACC-TAMRA (where FAM is 6-carboxyfluorescein, and TAMRA is 6-carboxytetramethylrhodamine). sgRNA-E-F, CGATCTCTTGTAGATCTGTTCTC; sgRNA-E-R, ATATTGCAGCAGTACGCACACA; sgRNA-E-probe, FAM-ACACTAGCCATCCTTACTGCGCTTCG-TAMRA. Viral loads were expressed on a log10 scale as viral copies per gram, after calculated with a standard curve. Viral copy numbers below the LLOD were set to half of the LLOD^46^.

### Histopathology analysis

Mouse lung tissues were fixed in 4% paraformaldehyde, dehydrated, embedded in paraffin, and sectioned. Tissue sections (4 μm) were deparaffinized in xylene and stained with H&E for pathological examination. To evaluate pathological changes in the lungs, the score was calculated as previously described^47^. The score was derived according to the following criteria: (1) no observable pathology; (2) perivascular infiltrates; (3) perivascular and interstitial infiltrates affecting < 20% of the lobe section; (4) perivascular and interstitial infiltrates affecting 20%–50% of the lobe section, and (5) perivascular and interstitial infiltrates affecting > 50% of the lobe section.

### ScRNA-seq

Single splenocytes were harvested at week 7 from vaccinated female 6–8-week-old C57BL/6 mice, which were administered a single dose of AdC6-S (day 0), AdC68-S (day 0), or AdC68-S + AdC6-S prime-boost vaccines (day 0, day 28), and mice administered adenovirus empty vectors were used as controls. In addition, single splenocytes were collected from a naive mouse as a blank control (*n* = 1). Single cells were captured and sequenced on an Illumina Nova Seq 6000, and the scRNA-seq library was prepared using the Chromium Single Cell 3′ Reagent Kit v3 (10× Genomics). Raw gene expression matrices were generated for each sample using the Cell Ranger (v.4.0.0) pipeline coupled with the mouse reference version mm10. The matrices were imported to R 4.0.3, and gene expression data analysis was performed using R packages/Seurat^48^. After filtration of doublets, cells from each capture were normalized using SCTransform normalization, and then multiple samples of the scRNA-seq dataset were integrated in Seurat. After integration, scaling, principal component analysis, and clustering were performed; the FindAllMarkers function in Seurat was used to identify markers for each of the identified clusters. Cell-type annotation was performed based on the respective clustering results combined with the R package singleR and expression of the known marker gene^49^. The UMAP method was used to visualize the single cells in 2nd embedding. DEGs and GO enrichment analyses were performed using R packages/clusterProfiler v3.16.1^50^. Monocle 3^51–53^ was used to infer the cell differentiation trajectory and illustrate the behavioral similarities and transitions. The expression matrix of follicular B cells derived from Seurat was used to build a CellDataSet for Monocle pipeline and partition the cells into supergroups after dimensionality reduction. Louvain community-grouped genes in the needed partition into modules were used and DEGs in some modules among samples were identified^54,55^. The plot cell module was used to plot the trajectory and color the cells by subcluster type or pseudotime.

### Statistical analysis

All results are presented as means ± SEM, and statistical analyses were performed using GraphPad Prism 8.0.1 (GraphPad Software). Comparison of data between groups was performed using one-way analysis of variance (ANOVA) with Tukey’s multiple comparisons test or Student’s *t*-test. Statistical significance was set at *P* < 0.05. The correlation between antibody titers and immune protection between total IgG titers and NT_50_ titers was analyzed using Pearson’s correlation analysis.

## Supplementary information

Supplementary Information

## Data Availability

The experimental protocols and the data analysis pipeline used in our work follow those outlined on the 10× Genomics and Seurat official websites and Monocle official websites. Custom scripts for analyzing the data are available upon request. Source data are provided in this study.
