# Supplementary material for: Heterologous prime-boost immunizations with chimpanzee adenoviral vectors elicit potent and protective immunity against SARS-CoV-2 infection
Source: Cell Discov. 2021 Dec 18;7:123. doi: 10.1038/s41421-021-00360-4 (PMC8684349; doi:10.1038/s41421-021-00360-4)
Supplement: Supplementary file 1 — Supplementary Information [file 41421_2021_360_MOESM1_ESM.pdf]

Supplementary information for

Heterologous Prime-boost Immunizations with Chimpanzee Adenoviral Vectors Elicit Potent and Protective Immunity against SARS-CoV-2 Infection

Jiaojiao Liu[1, 13], Kun Xu[2, 6,13], Man Xing[1, 13], Yue Zhuo[1, 13], Jingao Guo[3, 4, 13], Meng Du[1], Qi Wang[1], Yaling An[5], Jinhe Li[3,6], Ping Gao[3,6], Yihan Wang[1], Furong He[1], Yingying Guo[1], Mingxi Li[7], Yuchao Zhang[8,9], Linqi Zhang[7], George F. Gao[6,10,11]*, Lianpan Dai[2,3,10]*, Dongming Zhou[1,12]*

[1] Department of Pathogen Biology, School of Basic Medical Sciences, Tianjin Medical University, Tianjin, China.

[2] Key Laboratory of Tropical Translational Medicine of Ministry of Education, School of Tropical Medicine and Laboratory Medicine, The First Affiliated Hospital, Hainan Medical University, Hainan, China

[3] University of Chinese Academy of Sciences, Beijing, China.

[4] Institut Pasteur of Shanghai, Chinese Academy of Sciences, Shanghai, China.

[5] Savaid Medical School, University of Chinese Academy of Sciences, Beijing, China

[6] Research Network of Immunity and Health (RNIH), Beijing Institutes of Life Science, Chinese Academy of Sciences, Beijing, China

[7] Comprehensive AIDS Research Center, Beijing Advanced Innovation Center for Structural Biology, School of Medicine and Vanke School of Public Health, Tsinghua University, Beijing, China.

[8] CAS Key Laboratory of Tissue Microenvironment and Tumor, Shanghai Institute of

Nutrition and Health, University of Chinese Academy of Sciences, Chinese Academy of Sciences, Shanghai, China.

[9] School of Life Science, Fudan University, Handan Road 220, Shanghai, China.

[10] CAS Key Laboratory of Pathogenic Microbiology and Immunology, Institute of Microbiology, Chinese Academy of Sciences, Beijing, China

[11] Chinese Center for Disease Control and Prevention (China CDC), Beijing, China

[12] Shanghai Public Health Clinical Center, Fudan University, Shanghai,China

[13] These authors contributed equally: Jiaojiao Liu, Kun Xu, Man Xing, Yue Zhuo, Jingao Guo

* e-mail: gaof@im.ac.cn, dailp@im.ac.cn, zhoudongming@tmu.edu.cn

Supplementary Figure S1

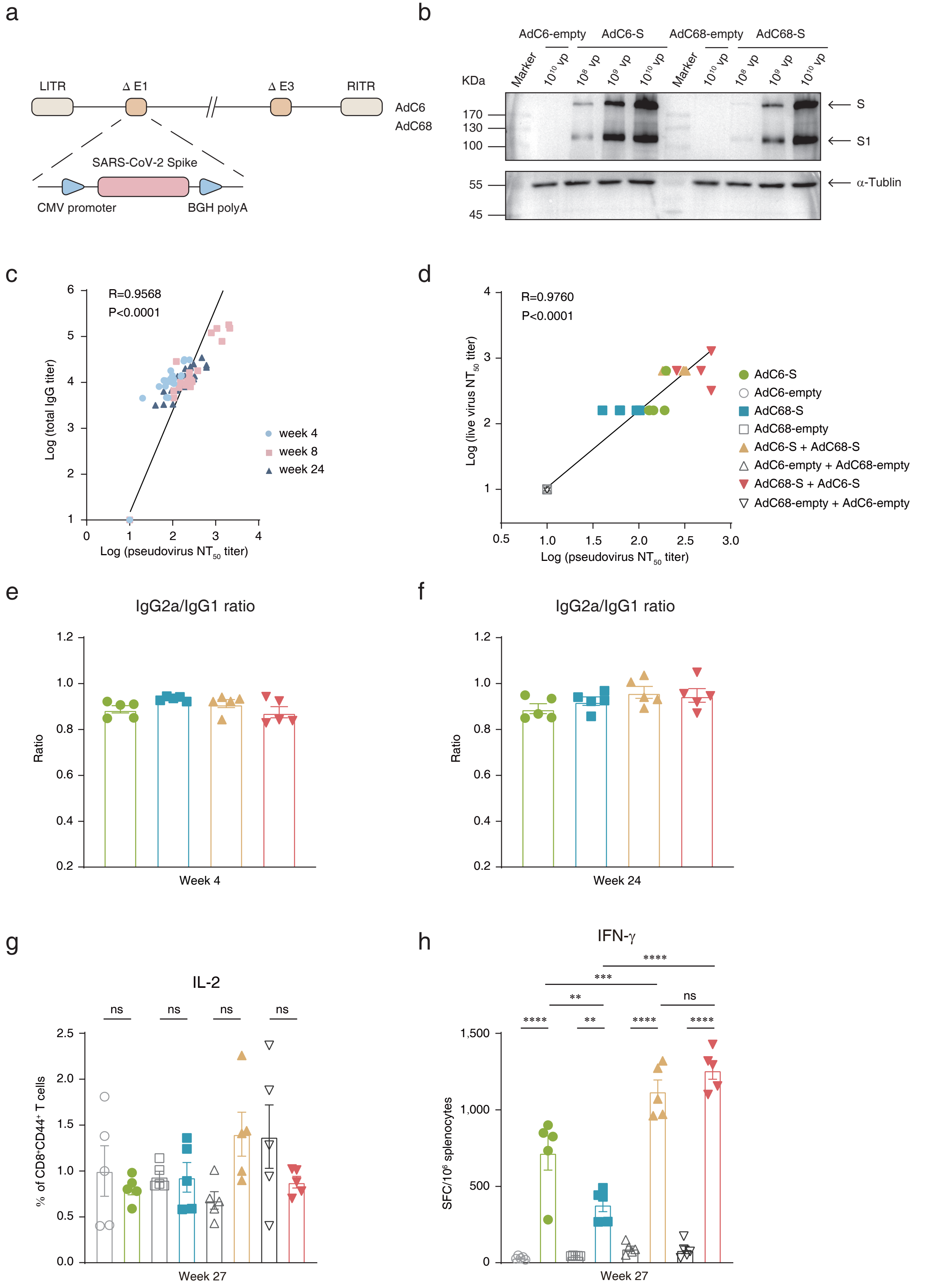

Supplementary Fig. S1 Construction and evaluation of recombinant AdC6 and AdC68 expressing full-length SARS-CoV-2 spike protein and immune response in vaccinated C57BL/6 mice. (a) Schema of AdC6 and AdC68 vaccines. The spike gene was inserted into the E1 region of AdC6 and AdC68 flanked by the CMV promoter and BGH polyadenylation signal sequence. (b) Western blot analysis of SARS-CoV-2 spike protein expression in HEK293 cells after infection with AdC6-S and AdC68-S (1×10^8, 1×10^9, and 1×10^{10} vp). AdC6-empty and AdC68-empty (1×10^{10} vp) were used as sham controls. The spike protein is present in both the cleaved and uncleaved forms; full-length spike protein (180 kDa) and S1 subunit (90 kDa) are marked. (c) Correlation of pseudovirus neutralizing antibody NT_{50} titers and spike-specific binding antibody titers and sera at week 4, week 8, and week 24 were collected. (d) Correlation between pseudovirus neutralizing antibody NT_{50} titers and live virus neutralizing antibody NT_{50} titers at week 24. Pearson's correlation was used for these analyses. (e–f) Endpoint titers ratios of IgG2a to IgG1 were calculated, mouse sera were collected at week 4 and week 24, and subtypes IgGs were assessed by ELISA. Mice sera immunized with AdC6-empty and AdC68-empty were not calculated, as the endpoint titers did not reach the lower limit of detection. (g) Intracellular cytokine staining was performed in mouse spleens to assess memory T cells at week 27, and cytokine IL-2 was detected. (h) ELISpot assays of IFN-γ secretion after stimulation with the S1 peptide pool, and cells were collected on week 27 post-vaccination. All data were displayed as mean ± SEM. *P*-values were analysed with one-way ANOVA (ns, $P\geq0.05$; *, $P<0.05$; **, $P<0.01$; ***, $P<0.001$; ****, $P<0.0001$).

Supplementary Figure S2

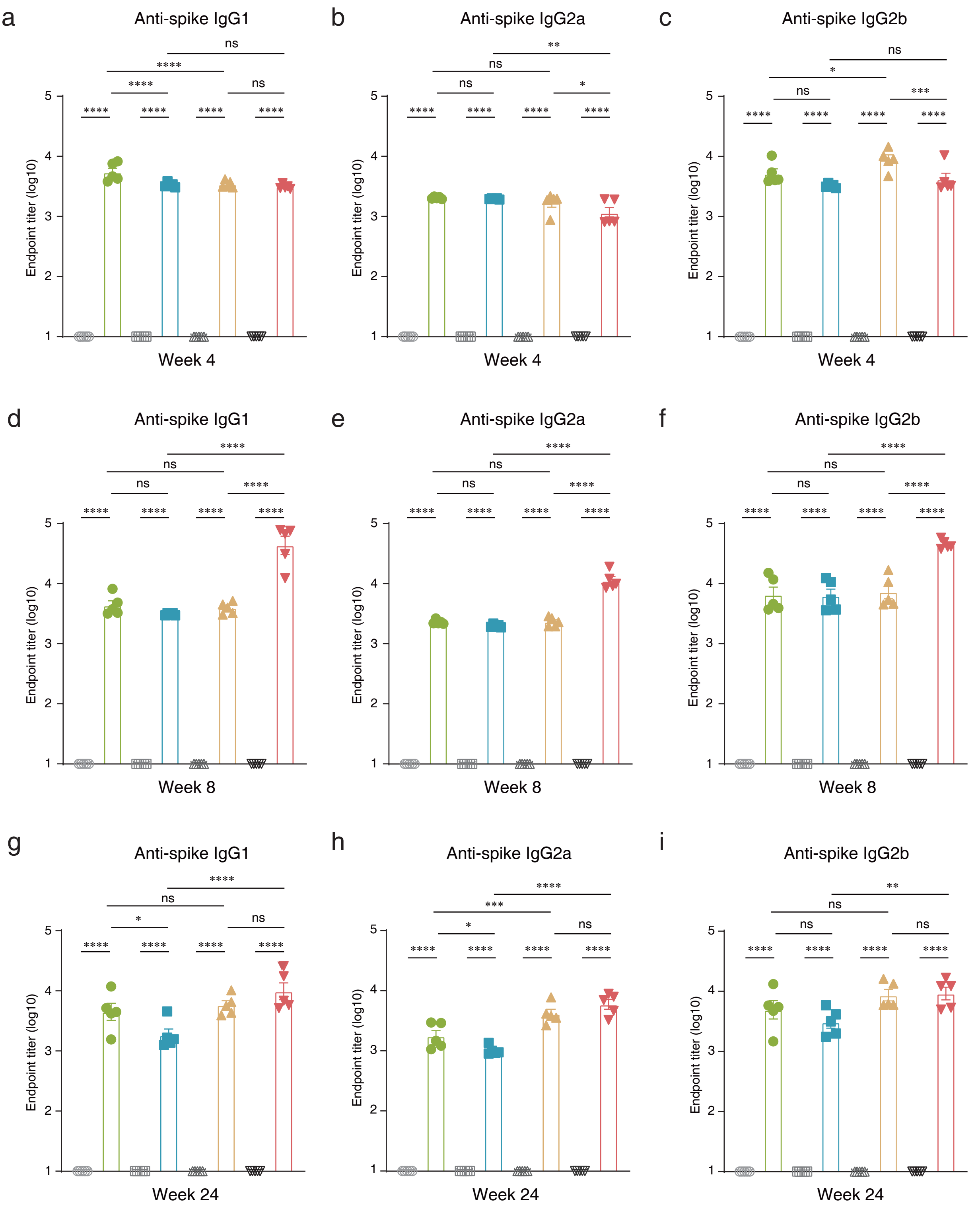

Supplementary Fig. S2 Spike-specific isotype IgG responses in vaccinated C57BL/6 mice (a–c) Represented reciprocal endpoint titers (log10) of spike-specific IgG1, IgG2a, and IgG2b at week 4 after intramuscular injection of vaccines, respectively. (d–f) Anti-spike-specific IgG isotype titers at week 8. (g–i) Anti-spike-specific IgG1, IgG2a, and IgG2b titers at week 24. All data were displayed as mean ± SEM. *P*-values were analysed with one-way ANOVA (ns, $P \geq 0.05$; *, $P < 0.05$; **, $P < 0.01$; ***, $P < 0.001$; ****, $P < 0.0001$).

Supplementary Figure S3

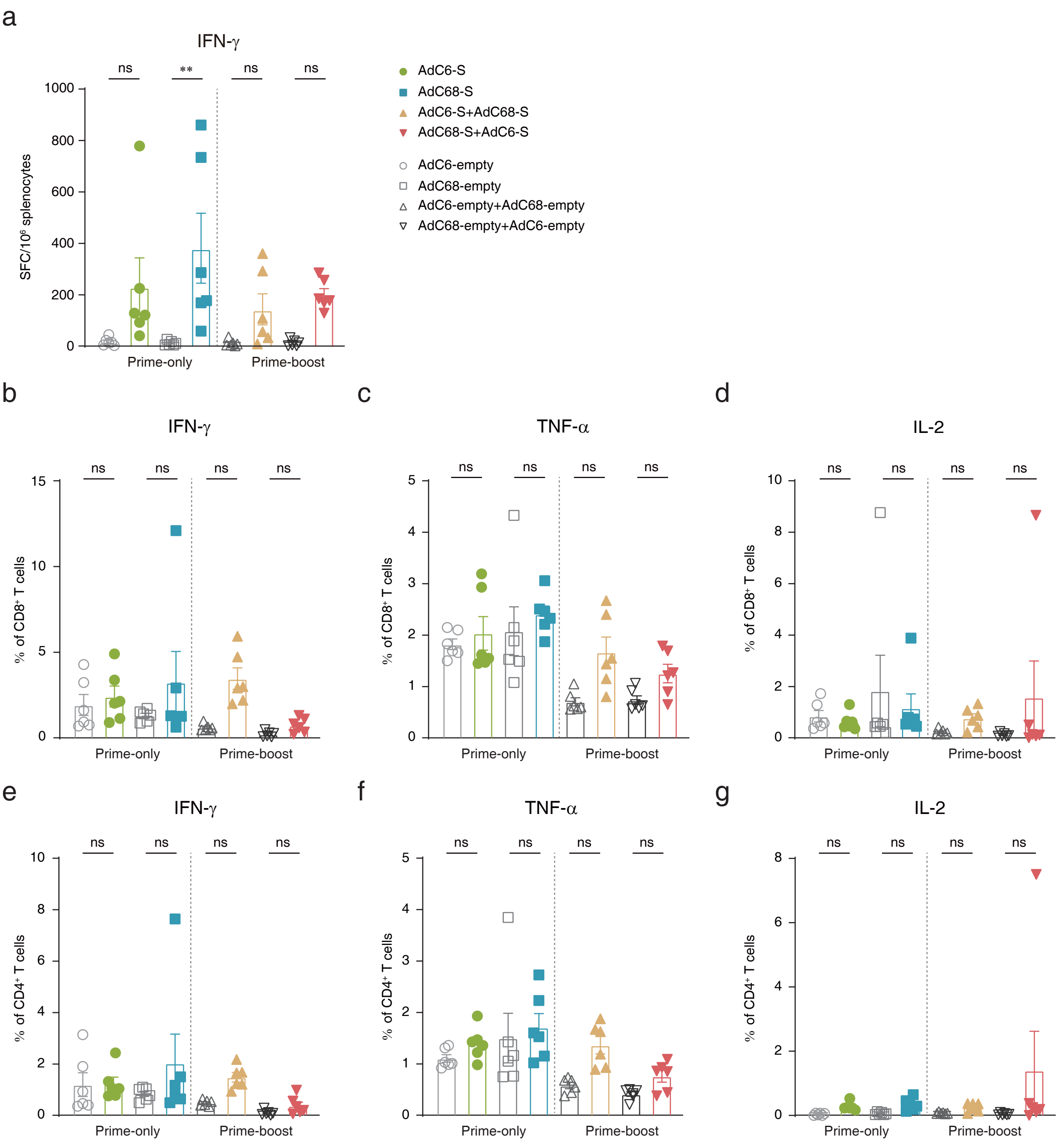

Supplementary Fig. S3 Cellular immune responses with stimulation with spike S2 peptide pool in vaccinated C57BL/6 mice. (a) ELISpot assay was performed to measure the IFN-γ secretion of splenocytes after S2 peptide pool stimulation in vaccine immunized mice. Splenocytes were harvested on day 10 post vaccinated. (b–d) Percentage of $CD8^+$ cytotoxic T lymphocytes expressing IFN-γ, TNF-α and IL-2 in response to the S2 peptide pool on day 10 post immunization. (e–g) Percentage of $CD4^+$ helper T cells expressing IFN-γ, TNF-α, and IL-2 in response to the S2 peptide pool on day 10 post immunization. All data were displayed as mean ± SEM. P-values were analysed with one-way ANOVA (ns, $P \geq 0.05$; *, $P<0.05$; **, $P<0.01$; ***, $P<0.001$; ****, $P<0.0001$).

Supplementary Figure S4

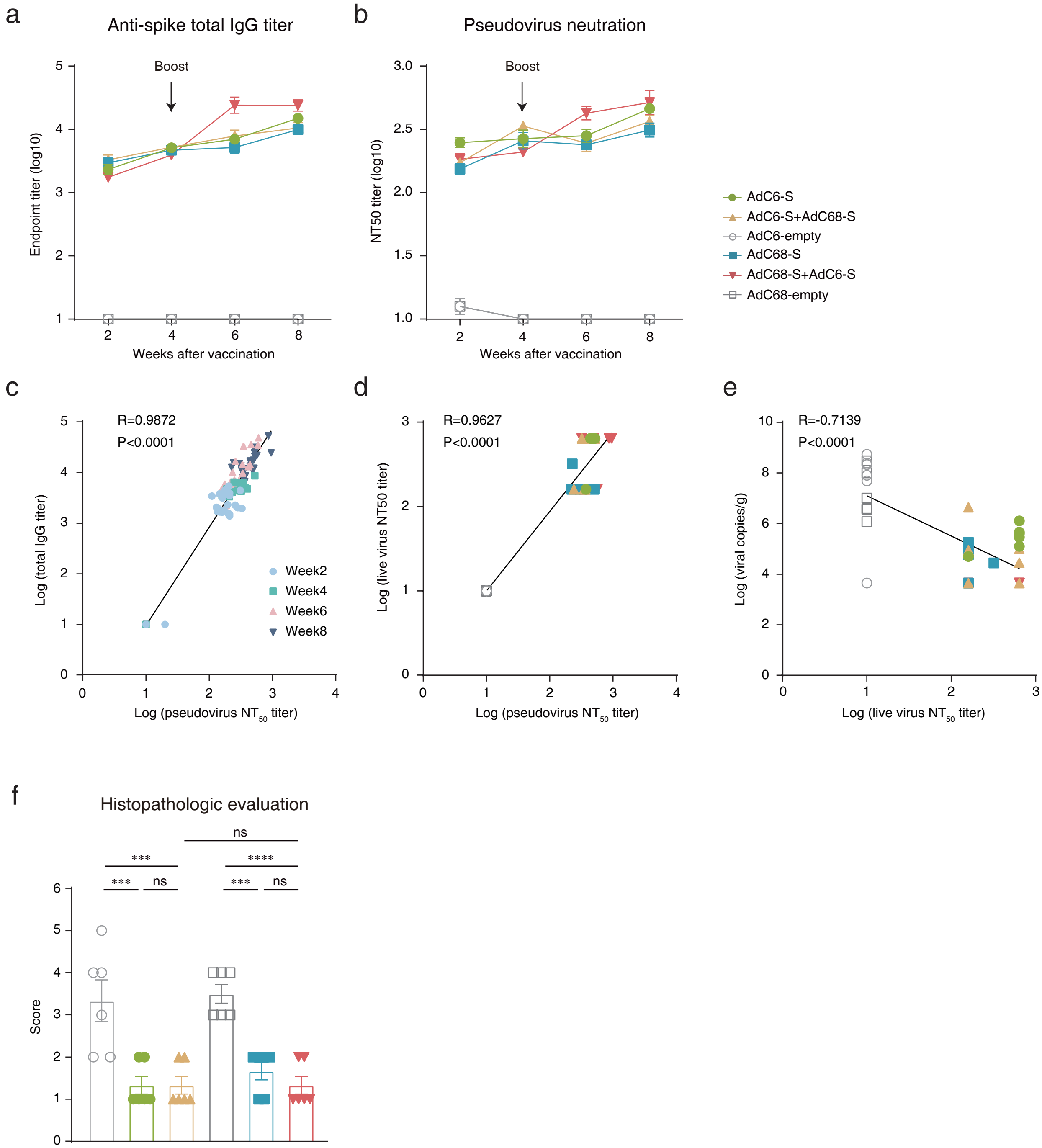

Supplementary Fig. S4 Characterization of humoral immune responses in BALB/c mice before SARS-CoV-2 challenge. (a) Anti-spike-specific binding total IgG endpoint titers of BALB/c mice (n=6) immunized with vaccine candidates via the i.m. route. (b) SARS-CoV-2 pseudovirus NT_{50} titers of serum samples from BALB/c mice (n=6) vaccinated with vaccine candidates. (c–d) Pearson's correlation of pseudovirus neutralizing antibody NT_{50} titers and spike-specific binding antibody titers or live virus NT_{50} titers. (e) Correlation of live virus NT_{50} titers prior to SARS-CoV-2 challenge and viral copies from lung tissues. The *P*-value was analysed using Pearson's correlation. (f) Histopathologic evaluation and scoring of viral lung infection. All data were displayed as mean ± SEM. *P*-values were analysed with one-way ANOVA (ns, $P \geq 0.05$; *, $P<0.05$; **, $P<0.01$; ***, $P<0.001$; ****, $P<0.0001$).

Supplementary Figure S5

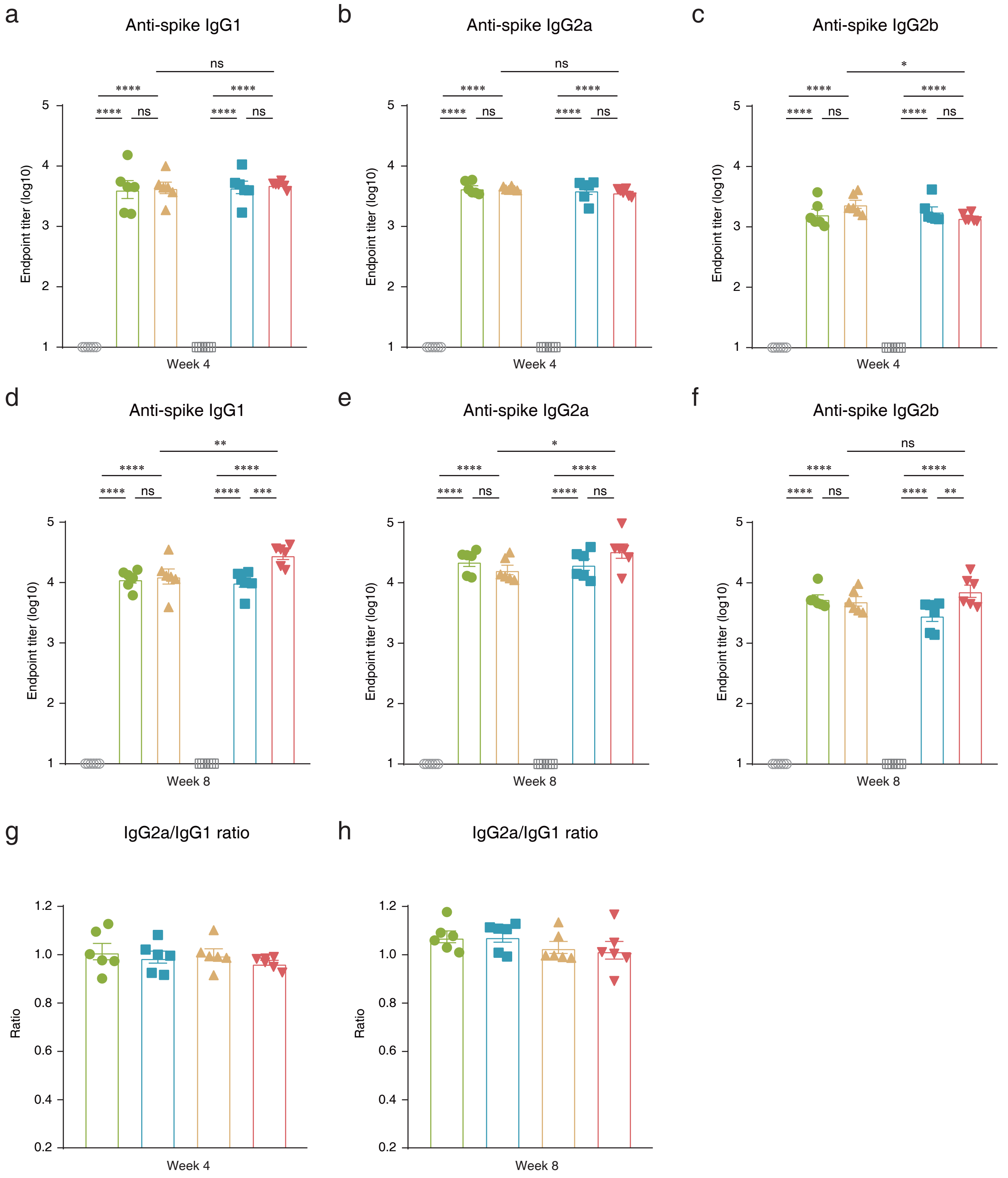

Supplementary Fig. S5 Spike-specific isotype IgG responses in vaccinated BALB/c mice (a–c) Reciprocal endpoint titers (log10) of spike-specific IgG1, IgG2a, and IgG2b of mouse serum samples collected at week 4 after intramuscular injection of vaccines. (d–f) Anti-spike-specific IgG isotype titers at week 8. (g–h) Endpoint titer ratios of IgG2a to IgG1 were calculated, mice sera were collected at week 4 and week 8, and mice sera of AdC6-empty and AdC68-empty were not calculated, for which endpoint titer did not reach the lower limit of detection. All data were displayed as mean ± SEM. *P*-values were analysed with one-way ANOVA (ns, $P \geq 0.05$; *, $P<0.05$; **, $P<0.01$; ***, $P<0.001$; ****, $P<0.0001$).

Supplementary Figure S6

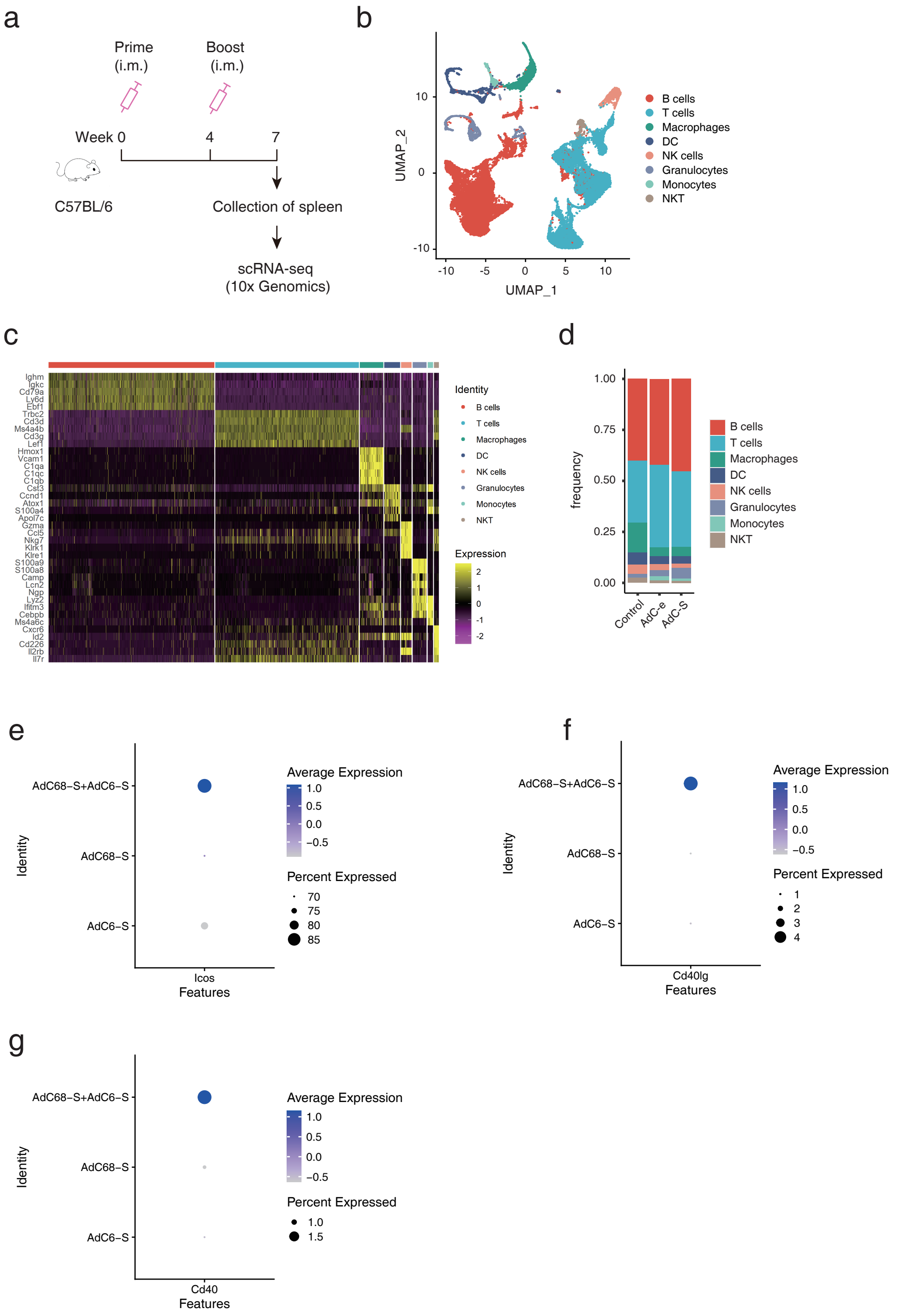

Supplementary Fig. S6 Single-cell RNA-seq of splenocytes from vaccinated C57BL/6 mice (a) Schedule of animal immunization and splenocyte collection. Batch one female C57BL/6 mice (6-8-weeks old) were immunized with 2 $\times 10^{10}$ vp of single-dose AdC6-S, AdC68-S, AdC6-empty, or AdC68-empty vaccines via the intramuscular route, respectively. Batch two mice primed with AdC68-S or AdC68-empty were boosted with 2 $\times 10^{10}$ vp of AdC6-S or AdC6-empty after four weeks of initial immunization (AdC68-S+AdC6-S, and AdC68-empty+AdC6-empty, respectively). Splenocytes were collected from one mouse of each group, and scRNA-seq was performed using 10× Genomics. (b) UMAP of splenocytes from vaccinated C57BL/6 mice. The cells are colored according to their lineage subtypes. (c) Heatmap showing the expression levels of cell-typing genes in subtype clusters. (d) Cell-type frequency in each group; bars are colored by cell type. (e) Bubble chart of the expression of *Icos* gene in effector CD4 $^{+}$ T cell cluster. (f) The expression of *Cd40lg* gene in effector CD4 $^{+}$ T cell cluster. (g) The expression of *Cd40* gene in follicular B cell cluster.

Supplementary Figure S7

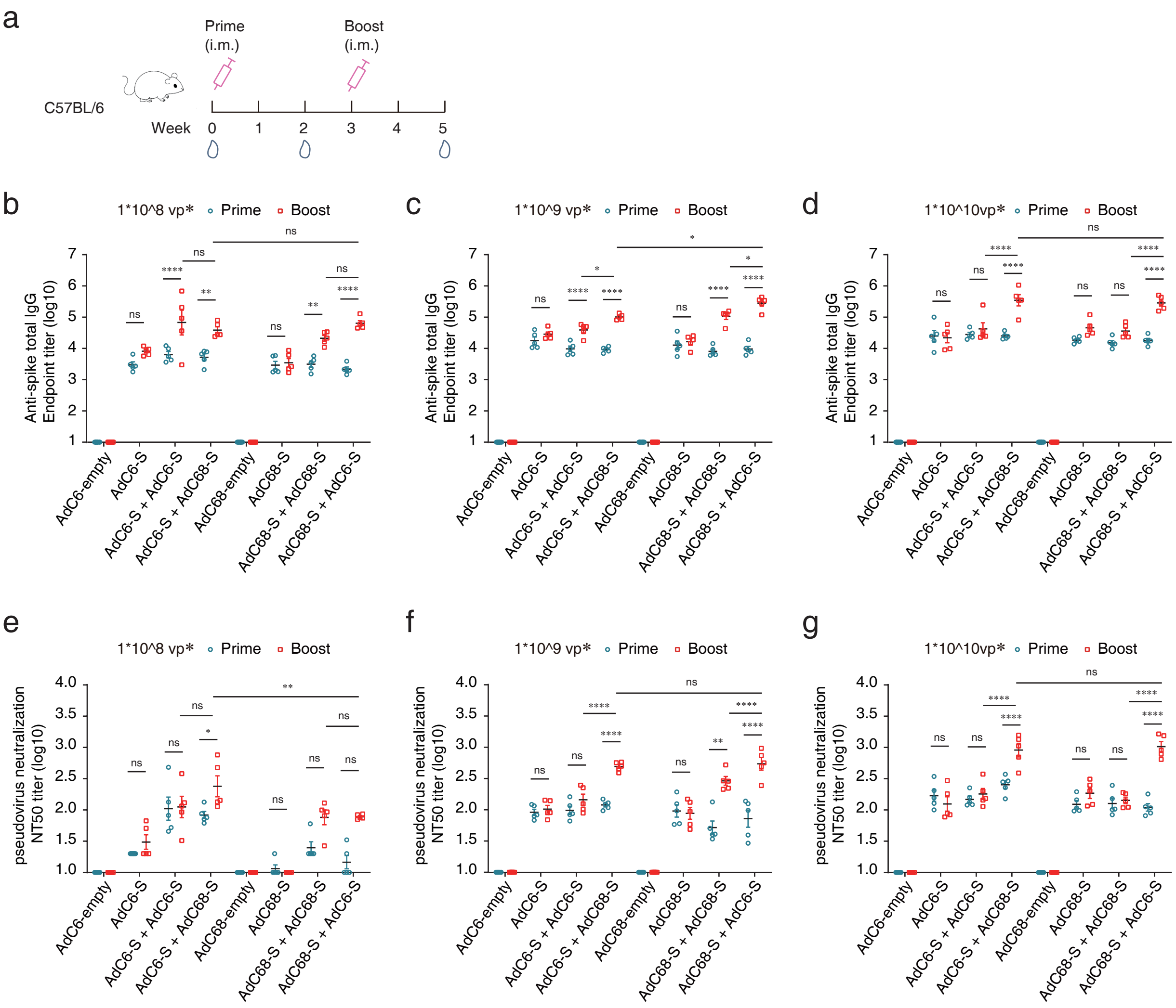

Supplementary Fig. S7 Antibody responses in vaccinated C57BL/6 mice. Adenoviruses AdC6-S (1.26 $\times 10^{10}$ ifu/mL) and AdC68-S (8.57×10^{9} ifu/mL) were re-amplified and purified (*), and the infectious units were titrated. 8-week-old female C57BL/6 mice (n=5) were vaccinated using prime-only regimen, homologous or heterologous prime-boost immunization, intramuscularly with 1×10^{8} vp (low dose), or 1×10^{9} vp (middle dose), or 1×10^{10} vp (high dose). (a) Schedule of chimpanzee adenoviral vaccine immunization and bleeding strategies. (b-d) Spike-specific total IgG reciprocal endpoint titers (log10) were measured using ELISA. (e-g) SARS-CoV-2 pseudovirus neutralizing antibody NT_{50} titers (log10) were measured. All data were displayed as mean ± SEM. *P*-values were analysed with one-way ANOVA (ns, $P \geq 0.05$; *, $P<0.05$; **, $P<0.01$; ***, $P<0.001$; ****, $P<0.0001$).

Supplementary Figure S8

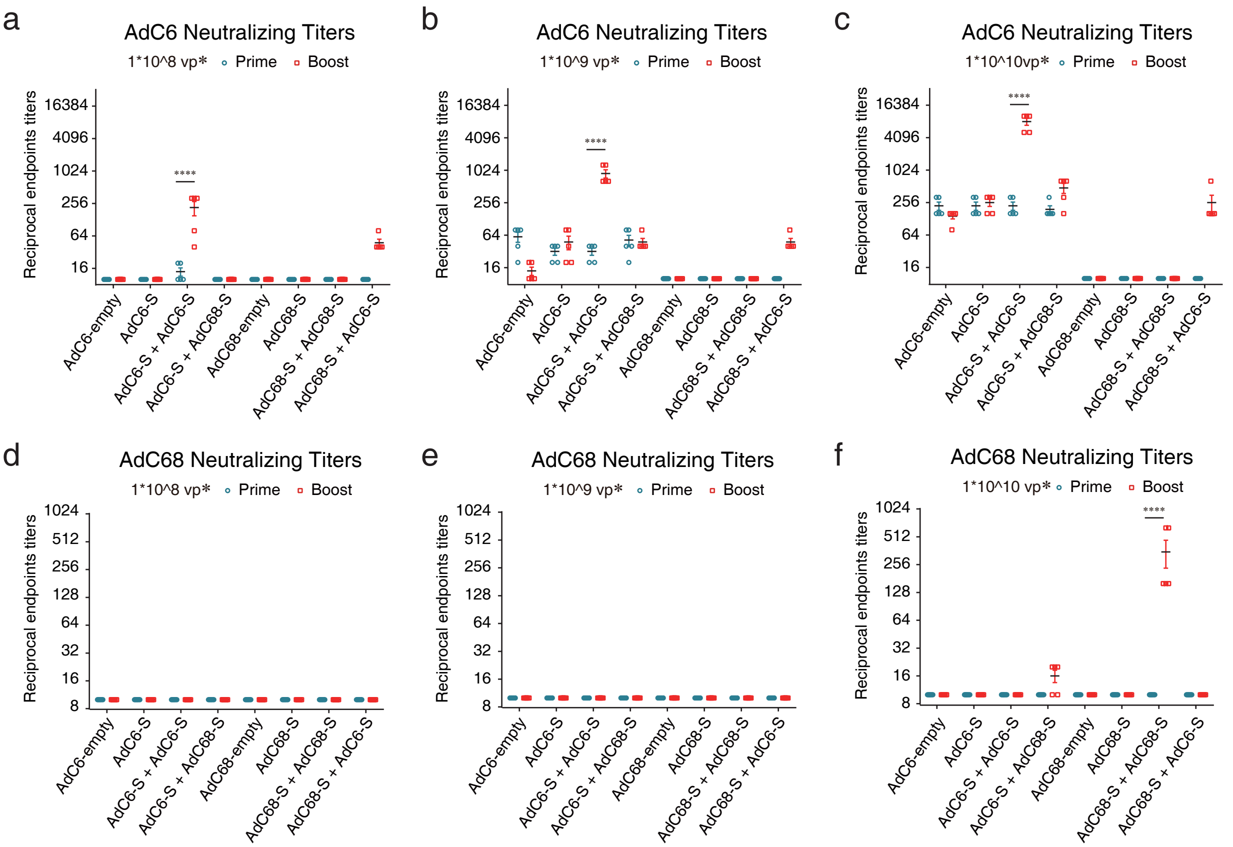

Supplementary Fig. S8 Adenovirus-specific neutralizing antibody responses in vaccinated C57BL/6 mice. Adenoviruses AdC6-S (1.26×10^{10} ifu/mL) and AdC68-S (8.57×10^{9} ifu/mL) were re-amplified and purified (*), and the infectious units were titrated. 8-week-old female C57BL/6 mice (n=5) were vaccinated using prime-only regimen, homologous or heterologous prime-boost immunization, intramuscularly with 1×10^{8} vp (low dose), or 1×10^{9} vp (middle dose), or 1×10^{10} vp (high dose). (a-c) AdC6-specific neutralizing antibody responses were presented. (d-f) AdC68-specific neutralizing antibody responses were presented. All data were displayed as mean ± SEM. *P*-values were analysed with one-way ANOVA (ns, $P\geq0.05$; *, $P<0.05$; **, $P<0.01$; ***, $P<0.001$; ****, $P<0.0001$).
